# Gateway-driven weakening of ocean gyres leads to Southern Ocean cooling

**DOI:** 10.1038/s41467-021-26658-1

**Published:** 2021-11-09

**Authors:** Isabel Sauermilch, Joanne M. Whittaker, Andreas Klocker, David R. Munday, Katharina Hochmuth, Peter K. Bijl, Joseph H. LaCasce

**Affiliations:** 1 Institute for Marine and Antarctic Studies, University of Tasmania, Hobart, Australia; 2 Department of Earth Sciences, Faculty of Geosciences, Utrecht University, Utrecht, The Netherlands; 3 Australian Research Council Centre of Excellence for Climate Extremes, University of Tasmania, Hobart, Australia; 4 British Antarctic Survey, Cambridge, UK; 5 Alfred Wegener Institute Helmholtz Center for Polar and Marine Research, Bremerhaven, Germany; 6 School of Geography, Geology and the Environment, University of Leicester, Leicester, UK; 7 Department of Geosciences, University of Oslo, Oslo, Norway

**Keywords:** Palaeoceanography, Palaeoclimate, Physical oceanography, Sedimentology, Tectonics

## Abstract

Declining atmospheric CO_2_ concentrations are considered the primary driver for the Cenozoic Greenhouse-Icehouse transition, ~34 million years ago. A role for tectonically opening Southern Ocean gateways, initiating the onset of a thermally isolating Antarctic Circumpolar Current, has been disputed as ocean models have not reproduced expected heat transport to the Antarctic coast. Here we use high-resolution ocean simulations with detailed paleobathymetry to demonstrate that tectonics did play a fundamental role in reorganising Southern Ocean circulation patterns and heat transport, consistent with available proxy data. When at least one gateway (Tasmanian or Drake) is shallow (300 m), gyres transport warm waters towards Antarctica. When the second gateway subsides below 300 m, these gyres weaken and cause a dramatic cooling (average of 2–4 °C, up to 5 °C) of Antarctic surface waters whilst the ACC remains weak. Our results demonstrate that tectonic changes are crucial for Southern Ocean climate change and should be carefully considered in constraining long-term climate sensitivity to CO_2_.

## Abstract (web summary)

The role of Southern Ocean gateways contributing to the Eocene-Oligocene climate transition is still debated. Here, the authors present high-resolution ocean simulations to show that gateways opening led to a reorganization of ocean circulation, heat transport and Antarctic surface water cooling.

## Introduction

During the Early Cenozoic, the Earth underwent one of the most fundamental global climate changes known in geological history, from hot Greenhouse conditions (~52–34 million years ago, Ma) to cold Icehouse conditions (<34 Ma)^1^. A long, gradual cooling from ~52 Ma culminated in a dramatic decline of global mean surface^2^ and deep^1^ ocean temperatures, the expansion of continent-wide glaciers in Antarctica^3,4^, and the start of a circum-Antarctic sea ice ecosystem^5^, around 34–33 Ma. Understanding the key mechanisms driving this transition remains difficult, as several potential triggering events occurred around this time period, and only sparse geological records exist, particularly for the Eocene–Oligocene transition (EOT, ~34 Ma). Two main processes have been proposed as key climate drivers: long-term decreasing atmospheric carbon dioxide (CO_2_) levels^4,6^ passing a critical threshold^3^, and the onset of a thermally isolating Antarctic Circumpolar Current (ACC), induced by the tectonic opening of both Southern Ocean gateways (Tasmanian Gateway, TG; and Drake Passage, DP)^7,8^. In addition to the main proposed driving processes, other processes are thought to provide crucial pre-conditions, or contribute additionally to the global cooling, including: Antarctic glacial expansion leading to enhanced westerlies, Southern Ocean deep-water formation and consequent benthic cooling^9,10^; and the initiation and/or strengthening of the Atlantic Meridional Overturning Circulation leading to deep-water formation and CO_2_ drawdown^11,12^.

Uncertainties remain for all hypotheses. Atmospheric CO_2_ concentrations declined from >1125 ppm^4^ during the early Eocene (52–48 Ma) to ~780 ppm^3,4,6^ around the EOT. However, a clear connection to the global cooling δ^18^O trend remains difficult due to significant gaps in the paleo CO_2_ record prior to the EOT^6^. The wide range of CO_2_ estimates from different proxy methods, which diverge up to 1100 ppm for the same geological time^6^, complicates this further. Additionally, the simulated CO_2_ threshold under which intermediate-sized ice sheets form, ranges from 560 to 920 ppm^13^, depending on the ice sheet and climate model configuration and their often poorly constrained boundary conditions^9^.

Similarly, uncertainties exist around the timing and role of the Southern Ocean gateways opening^14–17^. Estimates for opening of the tectonically complex DP range from 49^14^ to 17 Ma^15^. Although the timing of TG opening is better constrained^16^, uncertainties remain around its evolving paleodepth through the Late Eocene^17^. Further, the impact of the gateways opening on the reorganisation of Southern Ocean currents and Antarctic cooling is disputed^18^. The onset of an eastward ACC appears delayed by up to 4–23 million years (Myr) after the inception of Antarctic glaciation^17,19,20^; and its vigour is estimated to be weaker than today^19^. In addition, some Southern Ocean proxy data reveal an increase in sea-surface temperatures (SSTs) across the EOT^21^. It has been hypothesised that poleward heat transport in the East Australian Current was curtailed with the initiation of the ACC, and that the latter thermally insulated Antarctica^7,8^. But in low-resolution (nominally 2–3.5°) ocean models, poleward heat transport by warm subtropical currents was weak even prior to the opening of the gateways^18,22,23^.

However, recent studies demonstrate the importance of increased spatial resolution for realistic ocean flow simulations. High-resolution models (<1°) are less diffusive and permit ocean eddies which are responsible for a significant proportion of ocean heat transport^24^. In addition, they allow for an increasingly accurate representation of large-scale seafloor geometry and major bathymetric features, such as mid-ocean ridges, as well as small-scale seafloor roughness, formed by, e.g. fracture zones, seamounts and abyssal hills, which have a substantial influence on the large-scale ocean circulation, subsurface velocities and vertical structure of currents^25^. The bathymetric influence is particularly strong in the Southern Ocean^26^ where the ocean stratification is weak. These findings emphasise the importance of numerical resolution and seafloor roughness in simulations of the crucial EOT climate stage, to assess the oceanographic consequences and regional climatologic effects of different gateway depth configurations.

Our simulations accommodate both, high model resolution and seafloor roughness, to investigate the role of gateway opening on the Southern Ocean. Our study demonstrates that only small changes in the depths of the Tasmanian and Drake gateways are necessary to cause a dramatic reorganisation in ocean circulation pattern and Antarctic surface water temperatures. When at least one gateway is shallow (300 m), ocean gyres transport warm waters towards Antarctica. However, when this second gateway deepens below 300 m, the gyres weaken leading to a dramatic cooling of Antarctic surface waters (up to 5 °C) whilst the ACC remains significantly weaker than today.

## Results and discussion

### Approach

**Fig. 1:**
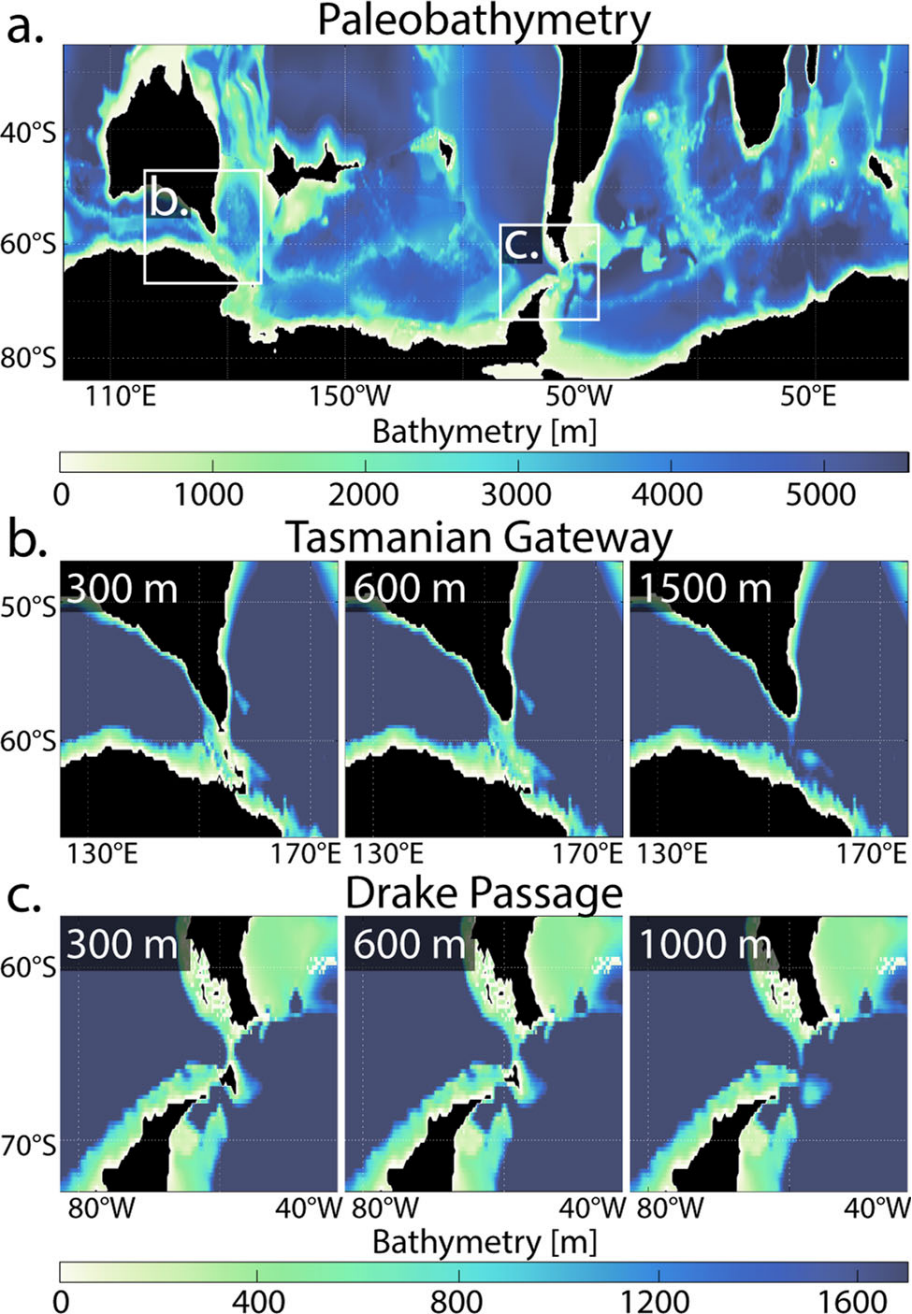
Paleobathymetry and gateway depths configurations. **a** High-resolution (0.25°) bathymetry of the Southern Ocean reconstructed to the Late Eocene (38 million years ago). **b**, **c** Stepwise subsidence of the (**b**) Tasmanian Gateway (TG) and (**c**) Drake Passage (DP), with depths adjusted to 300, 600 and 1000 m (DP) / 1500 m (TG). Black regions are above sea level.

In this work, we utilise increased available computational capabilities to run high-resolution ocean model simulations (0.25° horizontal grid spacing). While an ocean-only simulation does not allow for feedbacks between the atmosphere and ocean, it permits increased model resolution to resolve eddies and bathymetry. To complement this increase in model resolution, we employ a newly reconstructed paleobathymetry with detailed seafloor geometry and roughness^27^ (Fig. 1a) at higher resolution than in previous studies^18,22,23^. We simulate gateway-driven changes in ocean circulation and temperature distribution with a configuration of the Massachusetts Institute of Technology general circulation model (MITgcm) run with 50 vertical layers. We model a stepwise subsidence of both gateways, adjusting the depths to 300 m, 600 m and 1000 m (DP) / 1500 m (TG) (Fig. 1b, c). All model runs use the same forcing, taken from a coupled climate–ocean model^28^ under constant atmospheric CO_2_ concentrations representing Late Eocene conditions (800 ppm; see “Methods”).

### Southern Ocean was kept warm by ocean gyres

**Fig. 2:**
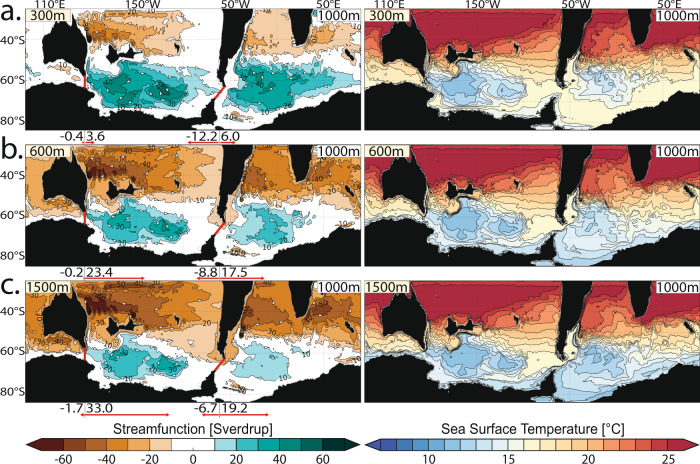
Impact of gateway deepening on Southern Ocean circulation and temperature. Oceanographic model results from progressive deepening of the Tasmanian Gateway (TG) from: **a** 300 m to **b** 600 m and **c** 1500 m water depths, with an already deep second gateway (Drake Passage (DP), 1000 m). See supplementary data for constant deep TG and progressively deepening DP. Left-hand side panels: ocean circulation patterns (annual mean and depth integrated stream function with contours indicating 10 Sverdrup intervals, white arrows indicate the flow direction). The zonal volume transport in east- and westward direction through both gateways (red lines) are indicated as red arrows and values in Sverdrup. Right-hand side panels: annual mean sea-surface temperatures (at 100 m water depth) with contours indicating 1 °C intervals. Black regions are above sea level.

When at least one gateway is shallow (300 m; Fig. 2a, Supplementary Fig. 3a), large-scale ocean gyres dominate the subpolar Pacific and Atlantic basins. These, the Ross and Weddell Gyres, are also present in previous modelling studies, e.g.^18,22,23^. The gyres are wind-driven and have transports exceeding 50 Sverdrups (1 Sv ≃ 10^6^ m^3^ s^−1^). In our model, they advect warm equatorial surface waters clockwise toward the Antarctic coast. As a result, SSTs reach 19 °C in the Australian-Antarctic Basin and 15–17 °C in the subpolar Pacific and Atlantic. The centres of the clockwise-circulating gyres are cooler (11–12 °C), due to (Ekman) upwelling of cold deep water. Our ocean model reproduces the warm pre-EOT conditions that have been proposed by the thermal isolation hypothesis^7,8^ and is largely consistent with the available proxy data showing warm southern high-latitude surface water conditions^21,29–34^. This stands in contrast to some previous models that could not reproduce the transport of warm water to the high Southern latitudes, and consequently simulated much colder pre-EOT Antarctic SSTs of ~2 °C, e.g.^18^.

**Fig. 3:**
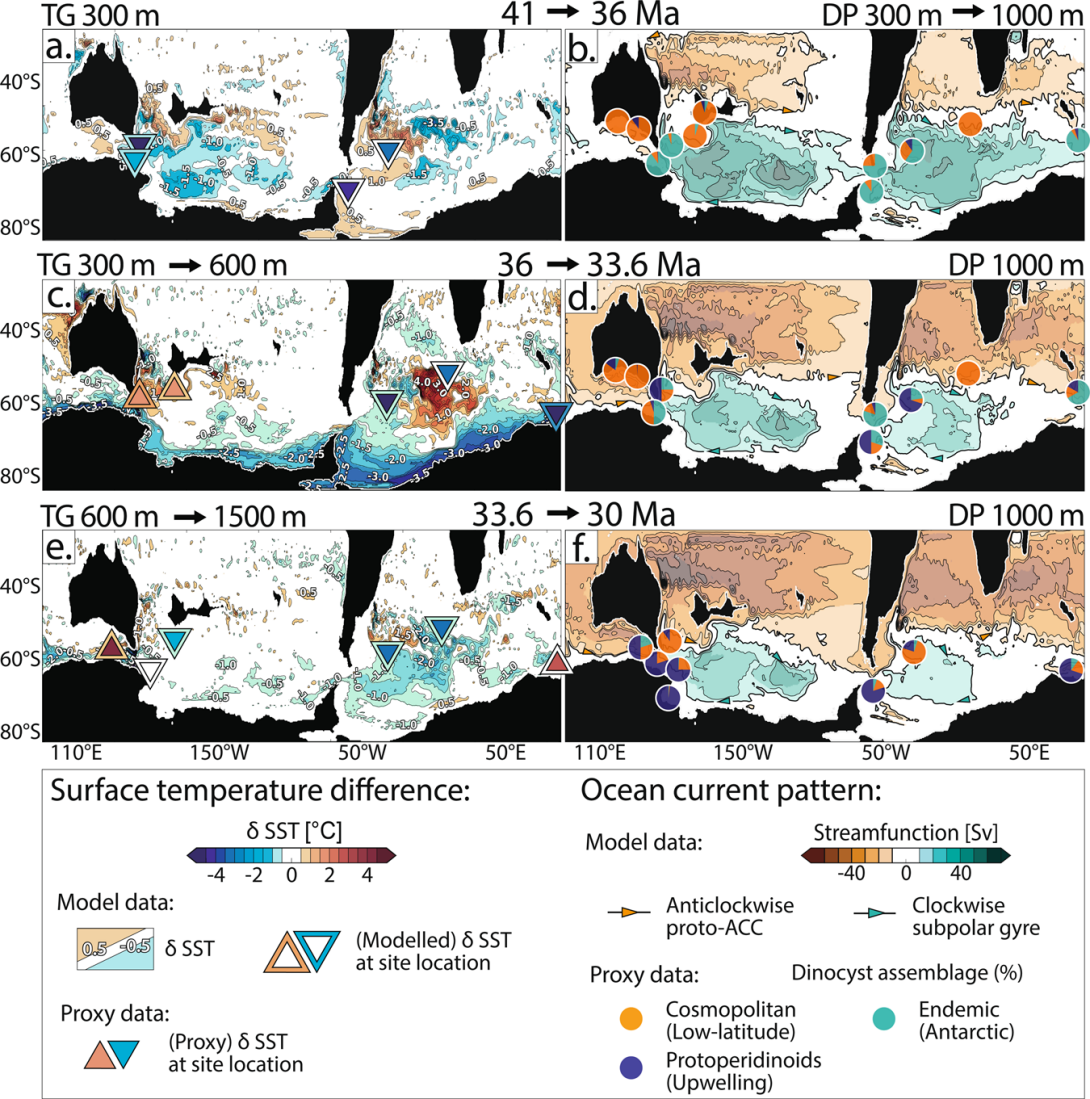
Paleoceanographic evolution of the Southern Ocean. **a**–**f** Model-data comparison and proposed paleoceanographic evolution of the surface Southern Ocean from the Late Eocene to Early Oligocene (41–30 million years ago, Ma). The left column presents the comparison of observed and modelled surface temperature differences (*δ* SST, both datasets use the same colour scale, see legend). Modelled differences in sea-surface temperatures result from the: **a** Drake Passage (DP) deepening from 300 to 1000 m (Tasmanian Gateway (TG) remaining at 300 m), TG deepening from: **b** 300 to 600 m and **c** 600 to 1500 m (DP at 1000 m). Contours indicate 0.5 °C intervals. The inner part of the triangles shows relative changes of paleo sea-surface temperature proxy records from sediment drill cores within the geological time slices^2,21,29–36,42,43^. The outer part of the triangles shows the modelled SST changes at these drill sites. The right column presents the comparison of observed and modelled current pattern. Modelled stream function pattern (bold contours show 10 Sverdrup (Sv) and −10 Sv; fine lines show >10 Sv and <−10 Sv; colour scale, see legend, ACC = Antarctic Circumpolar Current) are taken from simulations with: **b** TG at 300 m, **d** TG at 600 m and **f** TG at 1500 m (DP at 1000 m; Fig. 2). Pie charts present plankton biogeographic patterns in proportions (colours and affinities, see legend)^5,19,21,42–45^ found in sediment drill cores as proxy for surface current pattern. Details of the sites’ paleolocations, recorded geological time periods, as well as all data used in this study are collated in the SI.

Deepening of only one gateway (Tasmanian or Drake) does not cause significant changes in Southern Ocean circulations and SST distributions (Figs. 2a and 3a and Supplementary Figs. 3a, 6 and 9). Only small net transports through the TG and DP, of about 2–9% and 2–4% of today’s ACC transport (170 Sv^37^), respectively, are observed. A small proto-Antarctic counter current is detected flowing westward with velocities up to ~0.4 m s^−1^ (Supplementary Fig. 5).

### Dramatic cooling offshore Antarctica without a strong ACC

Once the second gateway subsides from 300 to 600 m, the subpolar gyres weaken (decreasing from >50 to >40 Sv). This leads to surface water cooling along the entire Antarctic coast (south of 60°S) between 2 and 3.5 °C, and extending up to 2000 km from the coast (Figs. 2b and 3c and Supplementary Figs. 3 and 4). Although the subpolar gyres are diminished, they still dominate the Southern Ocean circulation, with the net transport of circumpolar flow through the gateways approaching roughly 5–16% of the modern-day ACC. The proto-Antarctic counter current decreases in velocity and is largely replaced by the initiating, eastward flowing proto-ACC (Supplementary Fig. 5). Once the second gateway subsides below 600 m, the gyres weaken further to ~30 Sv, and the circumpolar current strengthens (~7–19% of today’s net transport). At the same time, the Antarctic coastal waters cool 0.5–2 °C further (Figs. 2c and 3e). The simulations show almost identical results, independent of which Southern Ocean gateway is the second one to deepen, TG or DP (Fig. 2 and Supplementary Figs. 3 and 4).

Our simulations show that gateway deepening enabled dramatic surface water cooling offshore the Antarctic coast without the initiation of a strong, fully developed ACC. Based on our results, we propose that the key oceanographic change caused by opening Southern Ocean gateways was the weakening of the Ross and Weddell gyres, rather than thermal insulation due to the onset of the strong proto-ACC. This driving mechanism resolves the long-standing conundrum of the delayed onset of a strong ACC, about 4–23 Myr after the EOT climate transition^17,19,20^. Using present-day bathymetry with the same paleo model forcing, the ACC transport was found to be about 22% that of the modern ACC (Supplementary Fig. 8). The increase in ACC strength to its present-day state was thus likely due to the decline of atmospheric CO_2_ and increase in the equator-to-pole SST gradients post-EOT (for details, see Supplementary Information, SI).

### Impact of model resolution and seafloor roughness

As noted, previous models did not capture the transport of warm water to the high Southern latitudes in the pre-EOT period and often exhibited colder Antarctic SSTs of ~2 °C, e.g.^18^. These models all employ ocean simulations with coarser resolution (nominally 2–3.5°). To examine this, we run additional simulations with coarser ocean model resolution (1°) and smoothed bathymetry (see SI, section 2.1.5). Our low-resolution model with one shallow gateway shows resulting subpolar gyres which are weaker and temperature advection which is significantly reduced, yielding colder SSTs along the Antarctic coast (Supplementary Fig. 12). The SSTs are also found to slightly increase in the south Pacific in response to deepening the TG, due to advection from the proto-ACC. A similar warming was observed previously in a 2° coupled simulation^38^. Increasing the resolution to 0.25° while retaining smoothed bathymetry yield strong subpolar gyres and temperature advection, but with markedly different spatial patterns than in the base simulations (Supplementary Fig. 13). Indeed, the transport exceeds 80 Sv in the eastern Ross Gyre, over a local depression in the smoothed bathymetry. We conclude that resolution is the primary factor for poleward heat transport, as the subpolar gyres are stronger. However, correct bathymetry is required to obtain realistic temperature distributions. As such, simulations employing present-day bathymetry^39^ should likewise yield quantitatively different SST distributions.

We note that our model is restored to prescribed surface boundary conditions^28^. As such, the temperature changes are effectively bounded. If, for example, the atmospheric heat transport were to increase to compensate for the reduction in oceanic heat transport^40,41^, the Antarctic could cool less. Conversely, having more zonally oriented SST gradients could reduce meridional atmospheric fluxes, enhancing the cooling. To address such feedbacks in detail, it is desirable to run coupled simulations, perhaps with a simplified atmosphere^22^, but with higher resolution in the ocean.

### Southern Ocean evolution: Model-empirical data comparison

Here, our aim is to model the consequences of gateways deepening on Southern Ocean circulation pattern and SST change. We do not expect our results to precisely match parameters such as absolute SST, but rather expect to observe matches in oceanographic circulation patterns and the magnitude of SST change. We look for agreement between modelled and observed patterns in (1) δ SST^2,21,29–36,42,43^, (2) plankton biogeography^5,19,21,42–45^ and (3) neodymium isotopic distribution^17,46^. We collate all available proxy data and group these data into three time periods between 41 and 30 Ma. Our model data with the different gateway depth configurations fit best with the proxy data as follows: at least one gateway remains shallow between ~41 and 36 Ma, the second gateway deepens from 300 to 600 m between 36 and 33.6 Ma, and further deepens (> 600 m) from 33.6 to 30 Ma (Fig. 3).

Overall, we find that our model results fit much of the available relative empirical oceanographic data from sediment drill cores in the Southern Ocean (Fig. 3).

### 41–36 Ma

Our simulation with at least one shallow gateway (300 m) exhibits strong gyres, warm Antarctic surface waters and small net transport through the gateways (Fig. 2a). These patterns are consistent with the Late Eocene (~41–36 Ma) plankton biogeography^42,44,45^ and SST proxy estimates^2,29–34,43^ (Fig. 3a, b).

With the first gateway deepening, our model shows a slight SST decrease in the central subpolar Pacific (−0.5 to −1.5 °C) and slight increase in the subpolar Atlantic (0.5–1 °C; Fig. 3a). Proxy SST data reveal general cooling at all drill sites located in the western subpolar Pacific and Atlantic basins (−3 to −7 °C). Although, the trends in model and proxy data are aligned at these locations, proxy data show stronger SST cooling compared to our model (Fig. 3a).

A key observation from the proxy records is the differences in SST values between the different ocean basins, but at similar latitudes^29^. Warmer subpolar Pacific SSTs (18–25 °C^2,29–32^) offshore Tasmania and Zealandia are recorded compared to the subpolar Atlantic SSTs (13.5–14 °C^29^) offshore the Antarctic Peninsula and Argentina. Our simulations show similar absolute SSTs and their differences between the major ocean basins, ~17–21 °C, offshore Tasmania and Zealandia; ~13–17 °C, offshore Antarctic Peninsula and Argentina (Fig. 2a).

Plankton biogeography between 41 and 36 Ma shows a high abundance of Antarctic-endemic species at drill sites offshore Tasmania, southern South America and Kerguelen Plateau^21,42–45^. Cosmopolitan, low-latitude species are dominant in regions farther north and in the Australian-Antarctic Basin (Fig. 3b). Our model with at least one gateway shallow shows strong subpolar gyres and indicates that their western boundary currents likely caused transport of Antarctic-endemic microfossils towards the mid-latitudes (Fig. 3b; for details, see SI). The drill sites that are not in the scope of the subpolar gyres contain cosmopolitan species, likely transported from the lower latitudes by subtropical gyres’ currents (e.g. Proto-Leeuwin Current into the Australian-Antarctic Basin).

A prominent shift in neodymium isotopic composition from Atlantic to Pacific endmember values (εNd shift from −7 to −5) occurs at the Agulhas Ridge offshore South Africa (Supplementary Fig. 2a, b) indicating the influx of Pacific water masses into the subpolar Atlantic from 41 Ma and changing its bottom water isotopic composition^46^. Our model shows a weak connecting eastward flow passing through the DP (eastward transport increases from 3.7 to 6 Sv; Supplementary Fig. 2a, b) and moving northward with the Atlantic subpolar gyre along the Argentinian coast towards the mid-latitudes. This current pattern is likely responsible for the recorded εNd shift at the Agulhas Ridge’s drill site located in the gyre’s pathway.

### 36–33.6 Ma

Between 36 and 33.6 Ma, drill sites offshore Argentina and Prydz Bay, East Antarctica, record a SST cooling of 2–8 °C (Fig. 3c), with absolute SSTs reaching 10 °C around Antarctica^21,30,33^. Our model results, for deepening of a second gateway from 300 to 600 m, show SST cooling offshore southern South America, Prydz Bay and offshore Antarctica generally, of 2–3.5 °C (Fig. 3c). The timing of this Southern Ocean cooling (reaching absolute proxy SSTs of ~10–18 °C^21,30,33^) coincides with the formation of continent-wide glaciers in Antarctica around 33.6 Ma^1^. Based on the similarities between modelled and proxy SST changes, we propose that it is likely that the deepening of the second gateway below 300 m caused ice-proximal ocean cooling and may have affected Antarctic temperature and precipitation conditions.

The same 300–600 m model also matches proxy data showing a SST warming of 2–3 °C east of the TG between 36 and 33.6 Ma^21^; our model simulates up to ~3 °C warming (Fig. 3c and Supplementary Fig. 4a). This paradoxical observation has been previously linked to the onset of warm Proto-Leeuwin flow through the TG^21^. Our results support this observation, as eastward flow transport increases through the TG (Fig. 2b), but also show that the Southern Ocean gyres weakened and shrank as the gateways deepened, with less local upwelling of cold deep water likely contributing to this local warming effect (Fig. 2b, c).

Our model results, with the second gateway deepening to 600 m, show weaker subpolar gyres and stronger transport through the gateways (Figs. 2b and 3d). These patterns are consistent with observations of Antarctic species distribution, which are still strongly present at drill sites along both gyre’s western boundaries, but low-latitude dinocysts percentages start to increase^21^ (Fig. 3d). Protoperidinioids dinocysts, which are indicators for strong upwelling, likely due to sea ice presence, start to appear at most sites during this time period^5,21^, supporting our observed dramatic SST cooling (Fig. 3c, d).

### 33.6–30 Ma

Between 33.6 and 30 Ma, proxy SSTs continue to decrease at subpolar Atlantic and Pacific drill sites (2–3 °C^29–33^, Fig. 3e). Our model results with the second gateway deepening to >600 m show similar SST cooling pattern in these regions, particularly in the subpolar Atlantic (~0.5–2 °C, Fig. 3e). In contrast, proxy SSTs increase by 3–4 °C, e.g.^33^ in the Australian-Antarctic Basin and Prydz Bay, East Antarctica, which can be observed very locally in our model (Fig. 3e).

Upwelling-loving (likely sea ice affine) protoperidinioids dinocysts strongly dominate most Southern Ocean drill sites from 33.6 Ma onwards^21,36,44^ (Fig. 3f), which aligns with our continuously decreasing SSTs, modelled when the second gateway deepens to >600 m. The ratio between Antarctic-endemic and low-latitude (cosmopolitan) species changes around 33.6 Ma, with low-latitude dinocysts becoming more present in the Southern Ocean sites after 33.6 Ma compared to the Antarctic-endemic counterpart (Fig. 3f). This pattern indicates further weakened northward transport by the subpolar gyres, and stronger circumpolar flow^21^, which is consistent with our model results where both gateways reach depths >600 m. The southern gyres weaken (Fig. 3f), and the transports of circumpolar flow increase from 5–16% (8.7–23.2 Sv, Fig. 2b) to 7–19% (12.5–31.3 Sv, Fig. 2c) of today’s value.

In contrast, Neodymium isotopes at drill sites east of the TG do not indicate ACC-type flow until 30 Ma^17^ (Supplementary Fig. 2; εNd shift from −4 to −5/−6, i.e. from Pacific to Atlantic-Indian endmember values); or as late as 23 Ma, according to some seismic stratigraphic constraints^20^. This offset is likely because some geological indicators, including Neodymium isotopes^17^ and stratigraphic hiatus formation^20^, record bottom water rather than surface water flows, so these proxies require a stronger net vigour to detect oceanographic changes, such as onset of ACC-type flow.

### Tectonics play a crucial role in the EOT climate transition

Overall, our results are consistent with proxy-based ocean flow and heat transport constraints and strongly suggest that tectonically induced oceanographic changes played a fundamental role in Earth’s key climate transition into the modern Icehouse world. Our simulations achieve these results due to high model resolution and realistic bathymetry, compared with previous lower resolution models with smoother bathymetry. These permit more vigorous oceanic heat transport and higher SSTs along the Antarctic coast, compared to low resolution simulations. Although declining CO_2_ concentrations in the atmosphere may still have been the final trigger for ice sheet growth on Antarctica, we thus demonstrate that tectonic changes play a key role in the Southern Ocean climate. We suggest that the regional tectonic setting was a crucial prerequisite to set the CO_2_ threshold for Antarctic glaciation, both at the inception of glaciation, as well as during later climate stages. Considering the important role of Antarctic-proximal SSTs for ice sheet behaviour, this outcome needs to be more carefully considered to define long-term sensitivity of past climate and ice sheets to atmospheric CO_2_.

## Methods

### Bathymetry reconstruction and modification of gateway depths

The paleobathymetry in this study is reconstructed for 38 Ma, using the plate tectonic model of Matthews et al. (2016)^47^ in a paleomagnetic reference frame^48^.

Bathymetry at latitudes south of 40°S is reconstructed following Hochmuth et al. (2020)^27^, using sediment backstripping^49^ with the software BALPAL^50^. The northern part of the grids (north of 40°S) uses the paleobathymetry of Baatsen et al. (2016)^51^. The transition between both grids is smoothed to avoid artificial jumps in the bathymetry. The maximum depth is set to 5500 m. We use an approach that reconstructs backwards in geological time, where sediment packages deposited since 38 Ma are removed from the present-day bathymetry^52^, the plates reconstructed to their paleopositions^47^, and sea level^53^ and dynamic topography^54^ changes are accounted for. Compared to forward modelling techniques^55^, this approach allows the preservation of realistic bathymetric features of seafloor roughness and small-scale, detailed geometry, such as fracture zones and seamounts, which are similar to the present day, within the resulting paleogrid. Recent studies have shown that these small-scale features with slopes steeper than 0.05° significantly affect subsurface eddy velocities and the vertical structure of ocean circulation patterns^25,56^.

For the backstripping method, sediment thickness information is derived from seismo-stratigraphic interpretations, using seismic reflection and drilling data in the Southern Ocean, e.g. ^57–60^. Identified key seismic reflectors are converted from two-way travel time into depth below seafloor utilising sonobuoy data and seismic reflection stacking velocities. Post-38 Ma sediments are backstripped whilst underlying sedimentary material is decompacted. Sediment decompaction is calculated using the relationship between porosity and burial depth^61^ for sand/silt in shelf and ooze in abyssal regions of the Southern Ocean. Isostatic rebound of the underlying crust resulting from the sediment removal is calculated after Airy’s law. Thermal subsidence through time is corrected, using the cooling model after Stein and Stein (1992)^62^, for oceanic crust and large igneous provinces, and McKenzie (1978)^63^, for extended continental crust. Changes in sea level^53^ and dynamic topography^54^ (Model 6) are included in our paleobathymetry calculation. Seafloor that has been subducted since the Eocene is incorporated into the grid by using data from global models (Nazca Plate^47^) and regional paleobathymetric reconstructions (Scotia Sea^64^).

The paleodepths of both gateways (TG and DP) are modified, whilst preserving the overall gateway geometries (Fig. 1b, c). Both gateways’ deepest points are set to 300 m, 600 m, 1000 m/1500 m (for DP/TG, respectively) following previously published pre-EOT and/or EOT depth approximations with TG at 300 m^16^, 600 m and 1500 m;^17^ and DP at 300 m^15^, 600 m^14^ and 1000 m^14^.

### Ocean model configuration

The ocean model used is the MIT general circulation model (MITgcm, e.g.^65^), version MITgcmUV checkpoint62x) in an ocean-only configuration with no sea ice. The model domain is circumpolar and extends between 84°S and 25°S in latitude. An additional simulation is presented with the model domain extending between 84°S and 0° (see model run in SI, section 2.1.2). The horizontal grid spacing is 0.25°, equivalent to a meridional grid spacing of ~27.8 km and a zonal grid spacing of ~3 to ~25 km at the southern and northern boundary, respectively. Two additional simulations are presented with the horizontal grid spacing of 1° (see model runs in SI, section 2.1.5). All model configurations use 50 vertical levels, ranging from 10 m at the sea surface to 368 m at the bottom.

The spatial and vertical resolution of our model simulations is higher than most model configurations previously used for similar studies, which generally have a horizontal resolution of 1–3.75° and are often coupled to the atmosphere^18,22,28,66^. Our higher resolution at 0.25° has the advantage that mesoscale eddies with length scales of ~10–100 km are partially resolved. These eddies play a key role in the ocean circulations, in particular in the transport of ocean heat^23,67–69^, and, therefore, their representation is crucial for this study. In addition, our model resolves small-scale bathymetry features and seafloor roughness more accurately, which significantly affect the ocean circulation patterns, as mentioned in the Methods - Bathymetry reconstruction section above. We demonstrate the consequences of ocean model resolution and seafloor roughness in the SI, section 2.1.5. We present 1° coarse ocean simulations which run with the rough bathymetry grid and two representative gateway depth configurations (TG at 300 m and 1500 m, DP at 1000 m; SI, section 2.1.5.2). In addition, we run two 0.25° ocean simulations, however, with smoothed bathymetries and the same depth configurations (SI, section 2.1.5).

Our 0.25° model uses a linear drag coefficient of 0.0011, a nonlinear equation of state^70^, a seventh-order advection scheme^71^ for temperature and salinity and the K-profile parameterisation^72^. No parameterisation is used for the advection and diffusion due to mesoscale eddies. Partial cells are used in the vertical for a more accurate representation of bathymetry. In the 1° simulations, eddies are parameterised using the parameterisation by Gent and McWilliams (1990)^73^ and an isopycnal diffusivity of 1000 m^2^ s^−2^.

**Fig. 4:**
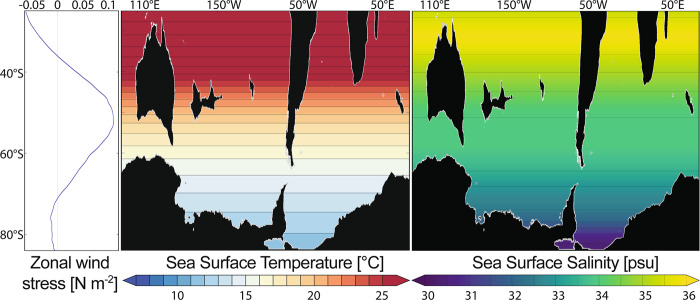
Restoring boundary conditions. Configuration of atmospheric forcing used in this model: stationary zonal wind stress (left), sea surface temperature (centre) and sea surface salinity (right); derived from a coupled ocean-atmosphere model simulation with atmospheric CO_2_ concentration of 800 ppm^28^, zonally and annually averaged.

Restoring boundary conditions for the surface forcing are taken from a coupled atmosphere-ocean model^28^ (GFDL CM2.1) simulating Late Eocene conditions with atmospheric CO_2_ concentrations of 800 ppm. The surface forcing for sea surface temperature, sea surface salinity, and zonal and meridional wind stresses are temporally and zonally averaged values from the coupled model (Fig. 4). The surface forcing is zonal averaged, to be able to modify the bathymetry configurations and continent-ocean distributions, without causing artificial disturbances of the ocean circulations. A sponge layer of ~300 km is used at the northern boundary of the model to relax to a temporal and zonal mean of salinity and temperature output from the coupled model with a restoring time scale of 10 days.

Each model simulation is spun up for 110 years. The results presented in the figures are time averaged over the last 10 years of the model run. The presented stream functions are calculated using depth integrated zonal velocities. The temperatures shown are from 100 m water depth.

### Model limitations

As a compromise between computational cost and realism of simulations we choose to run these simulations with an eddy-permitting horizontal resolution of 0.25°. This resolution only partially resolves mesoscale eddies, putting it into the space between fully parameterising or fully resolving eddies. This can lead to substantial numerical mixing and other numerical artefacts. However, this grid spacing resolution also allows for a more accurate representation of bathymetry, which we see as important for this study. The model simulations use an ocean-only configuration with SSTs and salinities restored to prescribed values. These restoring surface boundary conditions are likely to lessen changes observed between simulations since the restoring produces stronger temperature/salinity fluxes as SST/salinity differs more from the specified values. In addition, this model configuration does not include a sea-ice component.

## Supplementary information

Supplementary information.

## Data Availability

The model data generated, and the input data used in this study have been deposited in the IMAS data portal database with 10.25959/5eb21fc078c99. The bathymetry data used in this study are available in the IMAS data portal database with 10.25959/5eb222a378c9a.
