# Supplementary material for: Gateway-driven weakening of ocean gyres leads to Southern Ocean cooling
Source: Nat Commun. 2021 Nov 9;12:6465. doi: 10.1038/s41467-021-26658-1 (PMC8578591; doi:10.1038/s41467-021-26658-1)
Supplement: Supplementary file 1 — Supplementary information. [file 41467_2021_26658_MOESM1_ESM.pdf]

Supplementary Information for

Gateway-driven weakening of ocean gyres leads to Southern Ocean cooling

Nature Communications

Isabel Sauermilch[1,2*], Joanne M. Whittaker[1], Andreas Klocker[1,3], David R. Munday[4], Katharina Hochmuth[5,6], Peter K. Bijl[2], Joseph H. LaCasce[7]

[*] Corresponding author (contact: i.sauermilch@uu.nl)

[1] Institute for Marine and Antarctic Studies, University of Tasmania, Hobart, Australia.
[2] Department of Earth Sciences, Faculty of Geosciences, Utrecht University, Utrecht, The Netherlands.
[3] Australian Research Council Centre of Excellence for Climate Extremes, University of Tasmania, Hobart, Australia.
[4] British Antarctic Survey, Cambridge, United Kingdom.
[5] Alfred Wegener Institute Helmholtz Center for Polar and Marine Research, Bremerhaven, Germany
[6] School of Geography, Geology and the Environment, University of Leicester, Leicester, United Kingdom.
[7] Department of Geosciences, University of Oslo, Oslo, Norway.

Contents of this file

1. Geological proxy data from drill sites
2. Supplementary Information
   2.1 Additional simulations
2.1.1 Both gateways shallow (300 m)
2.1.2 Northern extension to the equator (Tasmanian Gateway: 300 m, Drake Passage: 1000 m)
2.1.3 Present-day bathymetry
2.1.4 Closed gateway (at 0 m)
2.1.5 Changing model resolution and seafloor roughness

1. Geological proxy data from drill sites

We compare the results of our simulations, of different depths of both gateways, to geological constraints in the Southern Ocean (Supplementary Figure 1) covering the geological time from Eocene to Early Oligocene (~41 to 30 Ma).

Geological sites which are located close to each other (50–300 km apart) and show similar microfossil species assemblages have been summarized into one data point in Figure 3. All details of the proxy records are collated in Supplementary Tables 1 and 2.

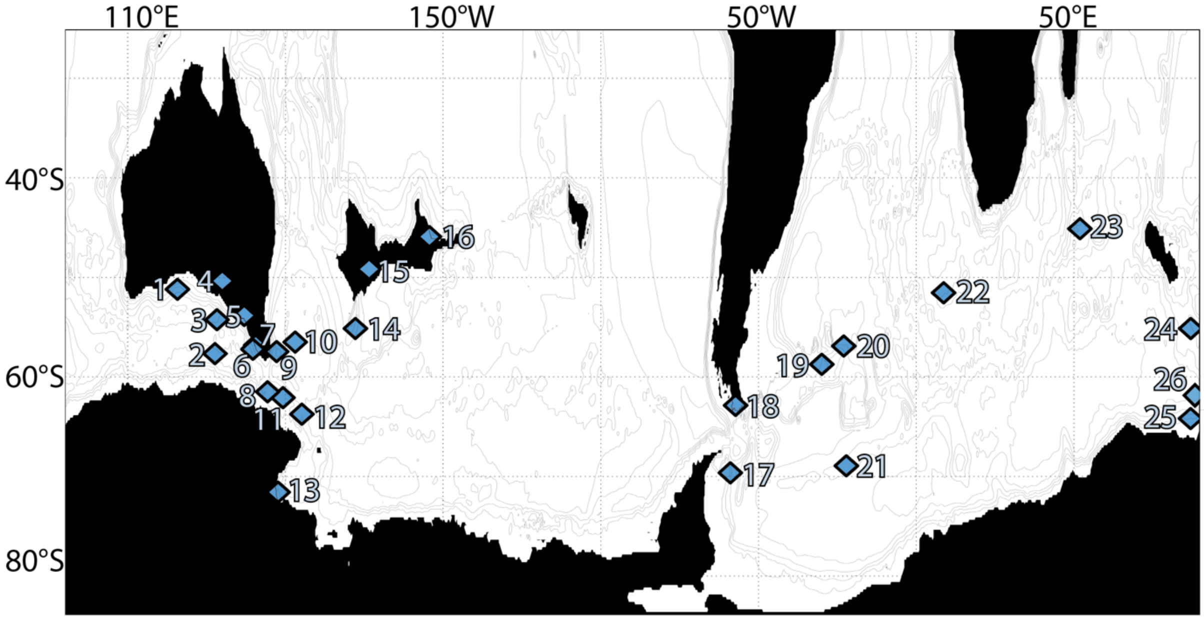

Supplementary Figure 1 Paleolocations (reconstructed at 38 Ma) of Southern Ocean sediment drill sites, where paleoenvironmental proxy data are taken and used for this study. All data are collated in Supplementary Tables 1–3.

1.1 Sea surface temperatures (SST) proxy records

SST data (Supplementary Table 1) are derived using two paleothermometer methods, tetraether index of 86 carbon atoms TEX_{86}[1] and alkenone unsaturation index $U^{k'}_{37}$[2,3]. Previously, it has been suggested that biomarker-based TEX_{86} proxies for Eocene SSTs may be biased towards warm temperatures in both polar regions[4,5], as seasonal biased export production of the biomarkers may have been dependent on the seasonality in grazing and fecal pellet formation[6]. However, this mechanism for a warm bias is not well-supported by

the available sediment trap studies. Although biomarker-based SST proxies tend to record warmer SSTs than other proxies, the SST trends per site are robust and are independent of the applied calibration[1].

We calculate our modelled SST differences from our simulations' absolute SST outputs between two gateway configurations. We compare these to the proxy SST changes within the respective geological time periods. The time period 41 to 36 Ma contains the Middle Eocene Climatic Optimum (MECO, ~40 Ma), a short-lived global warming event with global temperatures increasing by ~5 °C[7]. For studies presenting data including this anomaly[8], we removed this peak from the SST range presented in Supplementary Table 1 (noted with *).

Best fit between model and proxy data (Figure 3) is as followed: prior 41 Ma both gateways remain at ≤300 m; from 41 to 36 Ma one gateway deepens to >600 m depth; from 36 to 33.6 Ma, the second gateway transitions from 300 m to 600 m depth; and from 33.6 Ma onwards the second gateway deepens further (>600 m). Although, we do not compare modelled δ SST with proxy δ SST for the time period 49 to 41 Ma, we add the available proxy data for this particular time period to this overview for completeness.

Supplementary Table 1 Overview of absolute sea surface temperature proxy records and their relative changes (δ SST) within the geological time periods 49–41 Ma, 41–36 Ma, 36–33.6 Ma and 33.6–30 Ma, taken from Southern Ocean geological sites (see Supplementary Figure 1 for site location and referenced number).

Site	Label	Long (°)	Lat (°)	Paleo Long (°)	Paleo Lat (°)	Age span (Ma)	SST (°C)	δ SST (°C)	Method	Reference
**ODP 1172**	9	149.56	-43.58	157.21	-57.46	49-41	29-23	-6	TEX_{86}	Bijl et al. (2010)[9]; Bijl et al. (2013)[10]; Inglis et al. (2015)[11]; Douglas et al. (2014)[12]; Houben et al. (2019)[13];
						41-36	26-21	-5		
						36-33.6	21-23	+2		
						33.6-30	23	/		

**DSDP 277**	14	166.11	-52.13	-177.79	-55.13	49-41	13-10 *(?)*	-3	TEX_{86}, $U^{k'}_{37}$	Hollis et al. (2012)[14]; Douglas et al. (2014)[12]; Liu et al. (2009)[15]
						36-33.6	26-24	+2		
						33.6-30	24-22	-2		
**Waipara/ Hampden**	15	175.50	-41.40	-173.47	-49.18	49-41	27-20	-7	TEX_{86}	Hollis et al. (2012)[14]; Douglas et al. (2014)[12]; Inglis et al. (2015)[11]
**Seymour Island**	17	-56.17	-64.17	-58.94	-69.62	49-41	18-10	-8	TEX_{86}	Douglas et al. (2014)[12]; Inglis et al. (2015)[11]
						41-36	18-14	-4		
**DSDP 511**	19	-46.58	-51.00	-29.96	-58.73	41-36	25-22	-3	TEX_{86}, $U^{k'}_{37}$	Liu et al. (2009)[15]; Plancq et al., (2014)[16]; Douglas et al. (2014)[12]; Houben et al. (2019)[13]
						36-33.6	21-13	-8		
						33.6-30	13-10	-3		
**ODP 1170**	8	146.03	-47.09	154.34	-61.50	41-36	28-21 *	-7 * (without MECO -2)	TEX_{86}	Cramwinckel et al. (2020)[8]
**ODP 1090**	22	8.54	-42.55	8.71	-51.55	41-36	25	/	$U^{k'}_{37}$	Liu et al. (2009)[15]
						36-33.6	25-20	-5		
						33.6-30	20-17	-3		
**IODP U1356**	2	136.05	-63.35	137.70	-57.65	49-41	27-25	-2	TEX_{86}	Hartman et al. (2018)[17]; Bijl et al. (2013)[10]; Inglis et al. (2015)[11]
						33.6-30	18-22	+4		
**DSDP 274**	12	173.26	-68.60	165.24	-63.74	33.6-30	16-12	0	TEX_{86}	Hoem et al. (2020)[18]
**ODP 739, 742, 1166**	26	75.40	-67.55	87.49	-64.52	36-33.6	15-10	-5	TEX_{86}	Tibbett et al. (2021)[19]
						33.6-30	8-11	+3		

1.2 Dinocyst assemblage records

The biogeographic distribution of sedimentary dinocysts (Supplementary Table 2) are indicators for paleo surface water conditions: temperature[(20)], nutrient levels[(21)], salinity[(22)],

and upwelling, typically with presence of Southern Ocean sea ice[23,24]. The Paleogene Southern Ocean harbours strictly endemic dinocysts from Antarctic coastal regions, as well as a high abundance of bipolar dinocyst species that occur in both north and south polar regions[25-28]. Although the records of dinocyst assemblages show robust qualitative signals of surface currents connecting ocean basins, the water masses' vigour and vertical extension cannot be quantified from these microfossil assemblages.

In addition, dinocysts are transported spatially by ocean currents before settling down to the seafloor, and drill site. The travelled distances depend on several factors, including current speed, sinking speed of the particle and seafloor depth. Present-day modelling with particle tracing python module OceanParcels reveals that dinocysts (with an average sinking speed of 6 m/day) can be transported up to 1500 km in regions such as the strong Weddell Gyre[29]. No similar quantitative study exists for Eocene models. However, we follow Nooteboom et al. (2019)[30]'s study assuming meridional dinocyst transport of several hundred kilometres in the subpolar gyres (<1500 km transport due to the weaker gyre strengths compared to present-day; about 60–65 Sv, Weddell Gyre[29]; and 15-30 Sv, Ross Gyre[31,32]) and several hundred kilometres of zonal transport in regions dominated by circumpolar flow pattern. Endemic species are found in drill sites located along the gyres' western boundary currents, likely transported from the Antarctic coast to the mid-latitudes (Figure 3). Cosmopolitan species are likely transported zonally by a circum-polar flow; however, we assume less transport distance due to the weaker-than-today ACC transport (see main text, Figure 2). In order to quantify the exact transport distances, additional particle tracing experiments for the Eocene are required in the future.

Supplementary Table 2 Overview of dinocyst assemblages. Relative abundances of endemic-Antarctic, cosmopolitan/non-endemic, protoperidionioid (upwelling affine) species for the geological time periods 49–41 Ma, 41–36 Ma, 36–33.6 Ma and 33.6–30 Ma, taken from Southern Ocean geological sites (see Supplementary Figure 1 for site location and referenced number).

Site	Label	Long (°)	Lat (°)	Paleo Long (°)	Paleo Lat (°)	Endemic-Antarctic	Cosmopolitan / Non-endemic	Protoperidinioids (upwelling)	Reference
**49-41 Ma**									
ODP 1172	9	149.56	-43.58	157.21	-57.46	60	35	5	Bijl et al. (2011[28]; 2013[10])
DSDP 283	10	154.17	-43.55	163.08	-56.49	40	55	5	Bijl et al. (2011)[28]
ODP 1171	11	149.07	-48.30	159.21	-62.08	60	35	5	Bijl et al. (2011[28]; 2013[10])
Waipara	15	175.50	-41.40	-173.47	-49.18	5	90	5	Bijl et al. (2011)[28]
IODP U1356	2	136.05	-63.35	137.70	-57.65	75	25	0	Bijl et al. (2011[28]; 2013[10])
Punta Arenas	18	-68.78	-54.67	-57.29	-62.85	50	45	5	González-Estebenet et al., 2014[33]
DSDP 277	14	166.11	-52.13	-177.79	-55.13	0	100	0	Bijl et al. (2011)[28]
Seymour Island	17	-56.17	-64.17	-58.94	-69.62	75	15	10	Douglas et al. (2014)[12]
ODP 748	24	78.59	-58.26	87.03	-55.13	20	70	10	Bijl et al. (2011)[28]
Gippsland Basin	3	136.70	-38.30	138.32	-54.25	90	5	5	Bijl et al. (2011)[28]
Sorell Basin	7	145.02	-42.08	150.59	-56.81	0	100	0	Bijl et al. (2011)[28]
Otway Basin	5	143.18	-38.72	146.95	-53.82	0	100	0	Frieling et al. (2017)[27]
ODP 1170	8	146.03	-47.09	154.34	-61.50	90	10	0	Cramwinckel et al. (2018)[35]
**41-36 Ma**									
ODP 748	24	78.59	-58.26	87.03	-55.13	90	5	5	Bijl et al. (2011)[28]
Sorell Basin	7	145.02	-42.08	150.59	-56.81	0	90	10	Bijl et al. (2011)[28]
Browns Creek	4	138.65	-34.59	139.99	-50.36	5	95	0	Houben et al. (2019)[13]
Gippsland Basin	3	136.70	-38.30	138.32	-54.25	95	5	0	Bijl et al. (2011)[28]
ODP 1172	9	149.56	-43.58	157.21	-57.46	80	19	1	Bijl et al. (2011[28]; 2013[10])
ODP 1171	11	149.07	-48.30	159.21	-62.08	90	9	1	Bijl et al. (2011[28]; 2013[10])
ODP 1170	8	146.03	-47.09	154.34	-61.50	90	9	1	Cramwinckel et al. (2018)[35]
Moeraki Hampden	23	45.00	-45.00	51.89	-45.11	5	90	5	Cramwinckel et al. (2018)[35]
DSDP 277	14	166.11	-52.13	-177.79	-55.13	5	95	0	Bijl et al. (2011)[28]

Seymour Island	17	-56.17	-64.17	-58.94	-69.62	90	10	0	Douglas et al. (2014)[12]
Punta Arenas	18	-68.78	-54.67	-57.29	-62.85	75	25	0	González-Estebenet et al., 2014[33]
DSDP 512	20	-40.52	-49.52	-22.98	-56.89	60	30	10	Bijl et al. (2011)[28]
ODP 1090	22	8.54	-42.55	8.71	-51.55	0	100	0	Bijl et al. (2011)[28]
ODP 1168	6	144.25	-42.37	149.68	-57.22	0	95	5	Bijl et al. (2011)[28]
DSDP 511	19	-46.58	-51.00	-29.96	-58.73	50	25	25	Houben et al. (2019)[13]
ODP 696	21	-42.56	-61.51	-22.16	-68.93	90	5	5	Houben et al. (2019)[13]
ODP 1128	1	127.70	-34.50	125.77	-51.20	0	100	0	Bijl et al. (2011)[28]
**36-33.6 Ma**									
ODP 1172	9	149.56	-43.58	157.21	-57.46	25	25	50	Bijl et al. (2011[28]; 2013[10])
ODP 1171	11	149.07	-48.30	159.21	-62.08	50	45	5	Bijl et al. (2011[28]; 2013[10])
ODP 1170	8	146.03	-47.09	154.34	-61.50	75	10	15	Cramwinckel et al. (2018)[35]
ODP 1168	6	144.25	-42.37	149.68	-57.22	0	99	1	Bijl et al. (2011)[28]
Browns Creek	4	138.65	-34.59	139.99	-50.36	0	95	5	Houben et al. (2019)[13]
ODP 696	21	-42.56	-61.51	-22.16	-68.93	30	20	50	Houben et al. (2019)[13]
Punta Arenas	18	-68.78	-54.67	-57.29	-62.85	80	15	5	González-Estebenet et al., 2014[33]
ODP 1090	22	8.54	-42.55	8.71	-51.55	0	100	0	Bijl et al. (2011)[28]
DSDP 511	19	-46.58	-51.00	-29.96	-58.73	23	7	70	Houben et al. (2019)[13]
ODP 739	25	75.05	-67.17	87.01	-64.18	60	15	15	Bijl et al. (2011)[28]
ODP 1128	1	127.70	-34.50	125.77	-51.20	5	80	15	Bijl et al. (2011)[28]
**30 Ma**									
DSDP 511	19	-46.58	-51.00	-29.96	-58.73	5	75	20	Houben et al. (2019)[13]
ODP 696	21	-42.56	-61.51	-22.16	-68.93	5	15	80	Houben et al. (2019)[13]
DSDP 274	12	173.26	-68.60	165.24	-63.74	1	24	75	Hoem et al. (2020)[18]
CRP3	13	163.8	-76.9	157.67	-71.57	1	1	98	Houben et al. (2013)[23]
IODP U1356	2	136.05	-63.35	137.70	-57.65	0	20	80	Houben et al. (2013)[23]
ODP 739	25	75.05	-67.17	87.01	-64.18	10	20	70	Houben et al. (2013)[23]
ODP 1171	11	149.07	-48.30	159.21	-62.08	20	30	50	Bijl et al. (2011)[28]

1.3 Neodymium isotopic composition records

Water masses in each major ocean basin show characteristic signatures of Neodymium (Nd) isotope ratios (^{143}Nd/^{144}Nd, converted to εNd values; Supplementary Table 3) as a result of weathering of the ocean basin's surrounding geology. The εNd gradients between subpolar Pacific and Atlantic Ocean is characteristically steep prior the tectonic opening of the Southern Ocean gateways[36,37], as weathering of young volcanic West Antarctic rocks result in more radiogenic εNd values in the nearby Pacific Ocean seawater (-3 to -5[36,38]), whereas

the Atlantic Ocean's seawater shows less radiogenic values (about -9[36,39]), due to weathering of old East Antarctic cratonic material. εNd measurements on fossil fish teeth or ferromanganese crusts in sediments are used to track changes in bottom water masses, and potential interoceanic connections through the gateways[36,40].

Supplementary Table 3 Overview of neodymium isotopic composition records for the geological time periods 49–41 Ma, 41–36 Ma, 36–30 Ma and <30 Ma, taken from Southern Ocean geological sites (see Supplementary Figure 1 for site location and referenced number).

Site	Label	Long (°)	Lat (°)	Paleo Long (°)	Paleo Lat (°)	εNd (T)	Standard deviation (2δ)	Reference
**49-41 Ma**								
ODP 1090	22	8.54	-42.55	8.71	-51.55	-8.2 to -7.9	0.18-0.22	Scher and Martin (2006)[40]
**41-36 Ma**								
ODP 1090	22	8.54	-42.55	8.71	-51.55	-7.2 to -5.6	0.16-0.52	Scher and Martin (2006)[40]
**36-30 Ma**								
ODP 1090	22	8.54	-42.55	8.71	-51.55	-6.5 to -5.2	0.16-0.32	Scher and Martin (2006)[40]
ODP 1124	16	-176.53	-39.49	-154.33	-45.91	-5.0 to -3.9	0.1-0.31	Scher et al. (2015)[36]
ODP 1168	6	-144.25	-42.37	149.68	-57.22	-7.1 to -5.3 (36-33 Ma) -5.1 to -3.2 (33-30 Ma)	0.04-0.12	Scher et al. (2015)[36]
ODP 1172	9	-149.56	-43.58	157.21	-57.46	-4.8 to -3.3	0.09-0.3	Scher et al. (2015)[36]
**<30 Ma**								
ODP 1124	16	-176.53	-39.49	-154.33	-45.91	-6.2 to -5.4	0.04-0.15	Scher et al. (2015)[36]
ODP 1168	6	144.25	-42.37	149.68	-57.22	-7.2 to -6.1	0.05-0.23	Scher et al. (2015)[36]
ODP 1172	9	149.56	-43.58	157.21	-57.46	-6.8 to -3.6	0.08-0.29	Scher et al. (2015)[36]

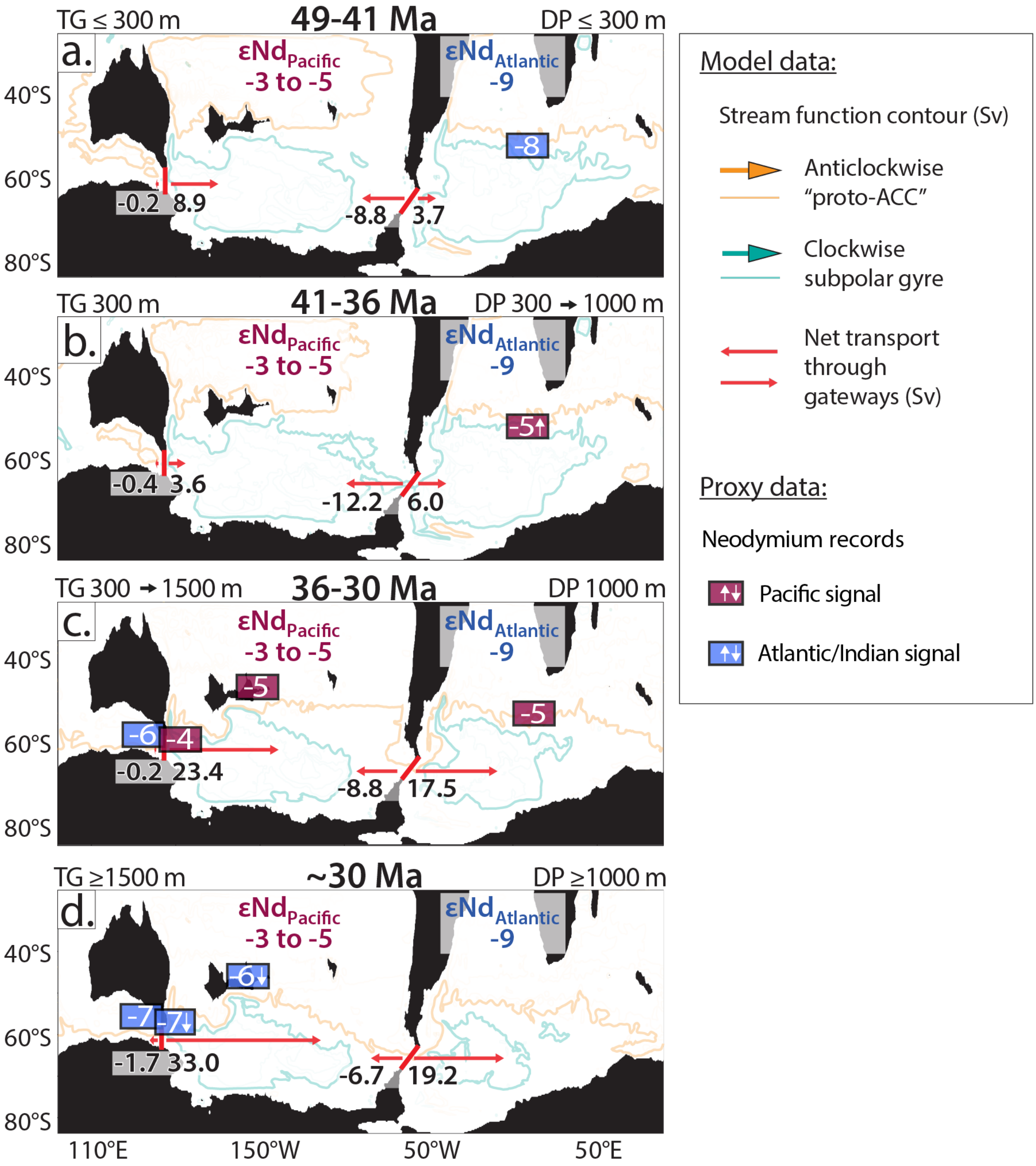

Supplementary Figure 2 Model-data comparison and proposed paleoceanographic evolution of the bottom Southern Ocean from the Eocene to Early Oligocene. The modelled stream function pattern (turquoise and orange lines represent stream function contours 10 Sv and -10 Sv) are taken from simulations with **a.** Tasmanian Gateway (TG) at 300 m, Drake Passage (DP) at 300 m, **b.** TG at 300 m, **c.** TG at 600 m, and **d.** TG at 1500 m (**b-d.** DP at 1000 m). The zonal volume transport in east- and westward direction through both gateways (red lines) are indicated as red arrows and values in Sverdrup. The squares show the neodymium isotopic composition records (numbers are absolute εNd values, arrows indicate increase/decrease

compared to the previous geological time slice)[36,40] as the proxy for bottom current flow direction (in comparison to Pacific endmember (purple square): -3 to -5[36,38], Atlantic endmember (blue square): -9[36,39]). Details of the sites' paleolocations, recorded geological time periods, as well as all data used in this study are collated in Supplementary Table 3.

2. Supplementary Information

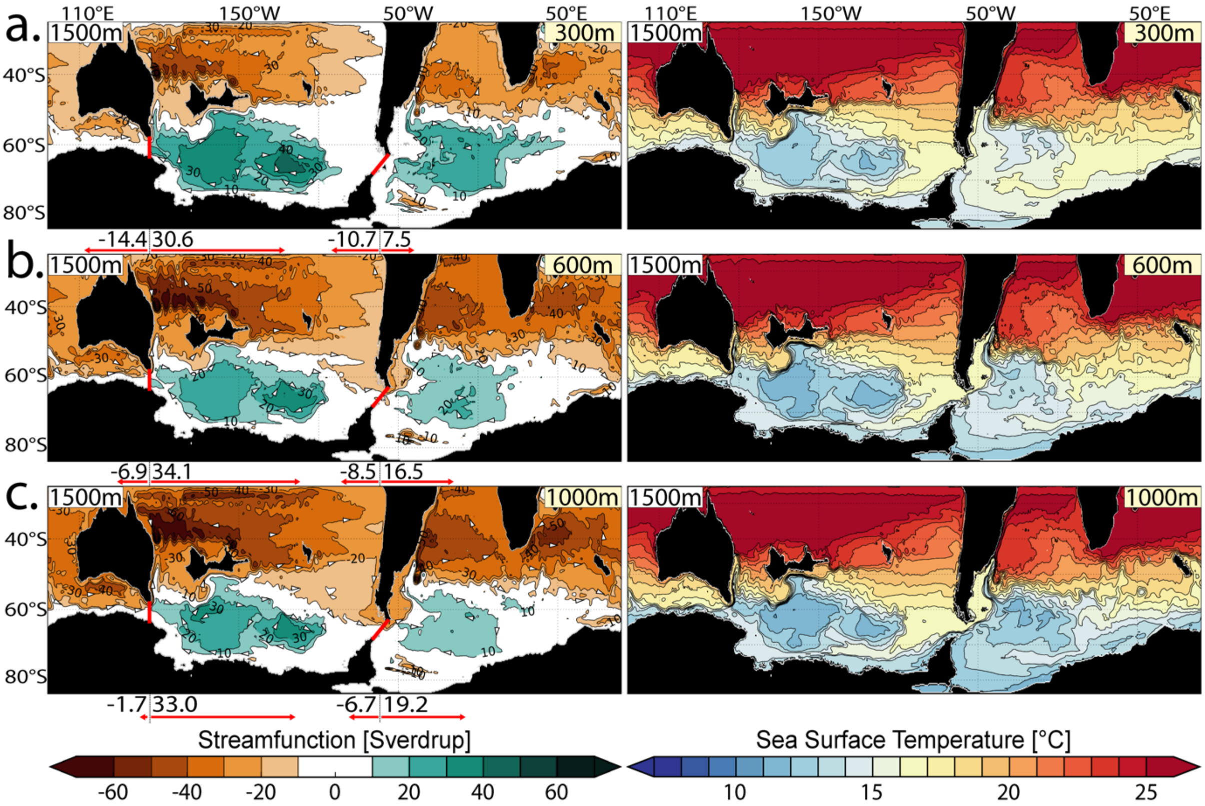

Supplementary Figure 3 Progressive deepening of the Drake Passage from **a.** 300 m to **b.** 600 m and **c.** 1000 m water depth (Tasmanian Gateway constant at 1500 m; legend as in Figure 2, main text).

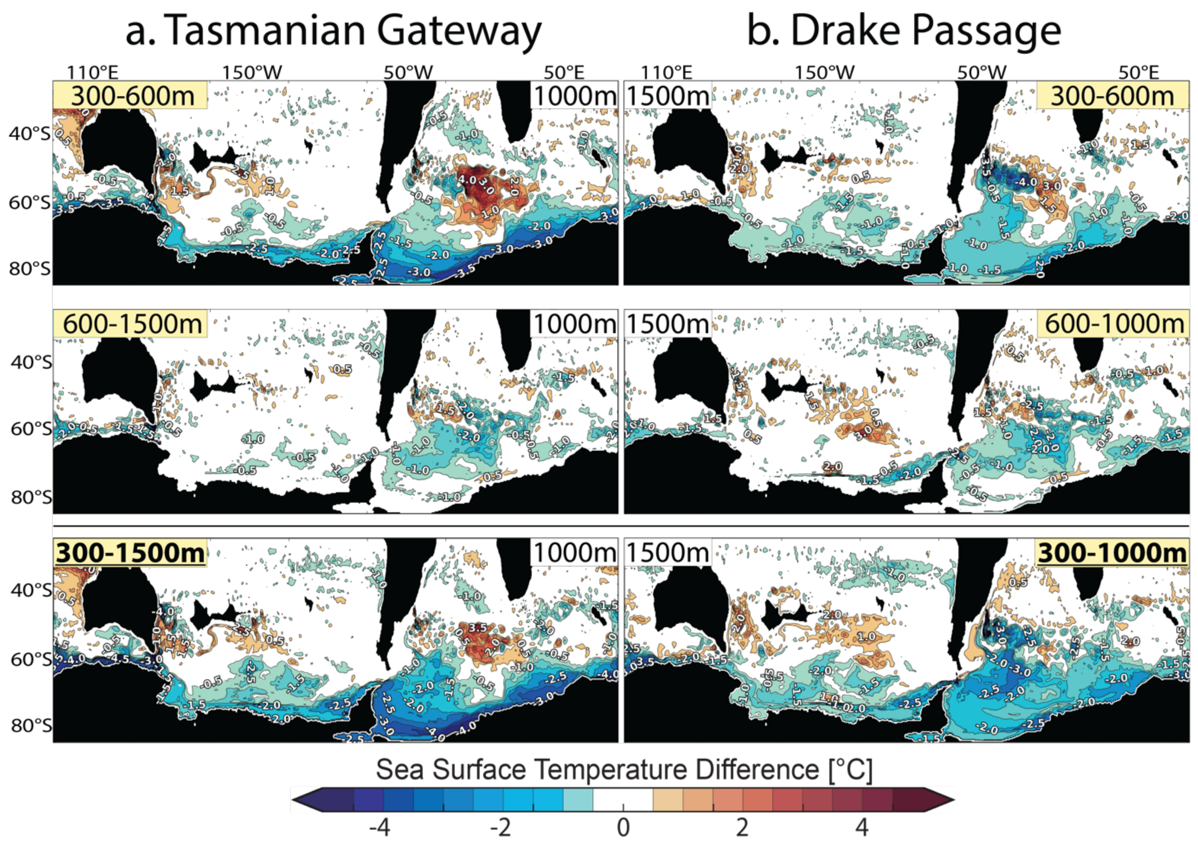

Supplementary Figure 4 Resulting SST differences after the deepening of **a.** the Tasmanian Gateway and **b.** the Drake Passage, from (1) 300 m to 600 m; (2) 600 m to 1500 m (TG) / 1000 m (DP); and (3) 300 m to 1500 m (TG) / 1000 m (DP). Contours indicate 0.5 °C intervals.

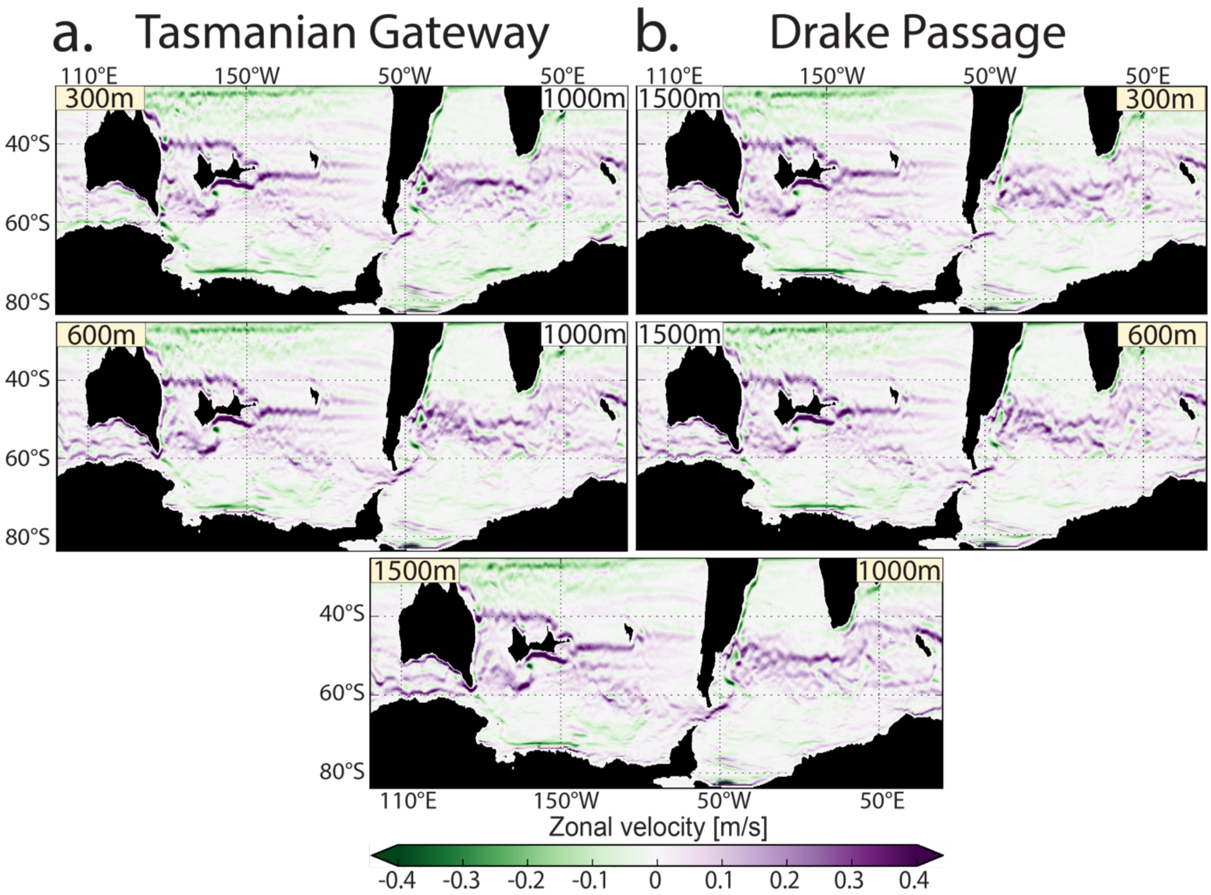

Supplementary Figure 5 Changing zonal velocity pattern at the surface (at 100 m water depth) resulting from progressive deepening of **a.** the Tasmanian Gateway from 300 m to 600 m and 1500 m water depths (Drake Passage, 1000 m); and **b.** the Drake Passage from 300 m to 600 m and 1000 m water depths (Tasmanian Gateway, 1500 m). Positive and negative velocities indicate east- and westward current velocity, respectively. Black regions are above sea level.

2.1 Additional simulations

Additional simulation results are presented for comparison, where 1) both gateways are 300 m deep, 2) the model's northern boundary is extended to the equator (with TG: 300 m, DP: 1000 m), 3) a present-day bathymetry grid (ETOPO-1[(41)]) is used, with paleo-forcing, 4) one gateway is entirely closed (0 m) and the second one is deep (1000 m), 5) the ocean model resolution is coarse (1°) and TG is 300 m and 1500 m deep (DP at 1000 m), and 6) the bathymetry is smoothed to 3° (TG at 300 m and 1500 m, DP at 1000 m) with high model resolution (0.25°).

2.1.1 Both gateways shallow (300 m)

The simulation with both Southern Ocean gateways at shallow depths (300 m) shows minor differences in ocean circulation and SST distribution to the models where one gateway has deepened (1000 m/1500 m). The net transports through the Drake Passage and Tasmanian Gateway are similar (3% and 5% of today's ACC transport (170 Sv[(42)]), respectively) to the net transports with one deep (>600 m) gateway (Figure 2a; Supplementary Figure 3a). However, the westward transport through each gateway is weaker than when this same gateway has deepened already (TG: -0.2 Sv versus -14.4 Sv, DP: -8.8 Sv versus -12.2 Sv; Figure 2; Supplementary Figures 3, 6).

The SSTs along the Antarctic coast are slightly warmer (1–2 °C) when both gateways are shallow, compared to the SSTs with a deep TG (Supplementary Figure 6b). Some regional warming effect is observed along the West Antarctic coast when the DP deepens from 300 m to 1000 m (TG: 300 m, Supplementary Figure 6b).

When one gateway is at shallow depth and the other one deepens from 300 m to 1000 m or 1500 m, a decrease in SST of up to 3 °C is observed in large areas of the Southern Ocean, particularly in the Pacific sector (Supplementary Figure 6b). This result is consistent with decreasing SST in proxy data at several Pacific drill sites during the Middle-Late Eocene (see Figure 3a, main chapter). This initial cooling event may be linked to the early ice sheet expansions reaching the Antarctic coast in several locations during this time period([43,44]). However, it remains speculative if such SST cooling event would have been sufficient enough

to trigger this large-scale pre-EOT ice sheet expansion, in comparison to the declining atmospheric CO_2 concentrations around this time.

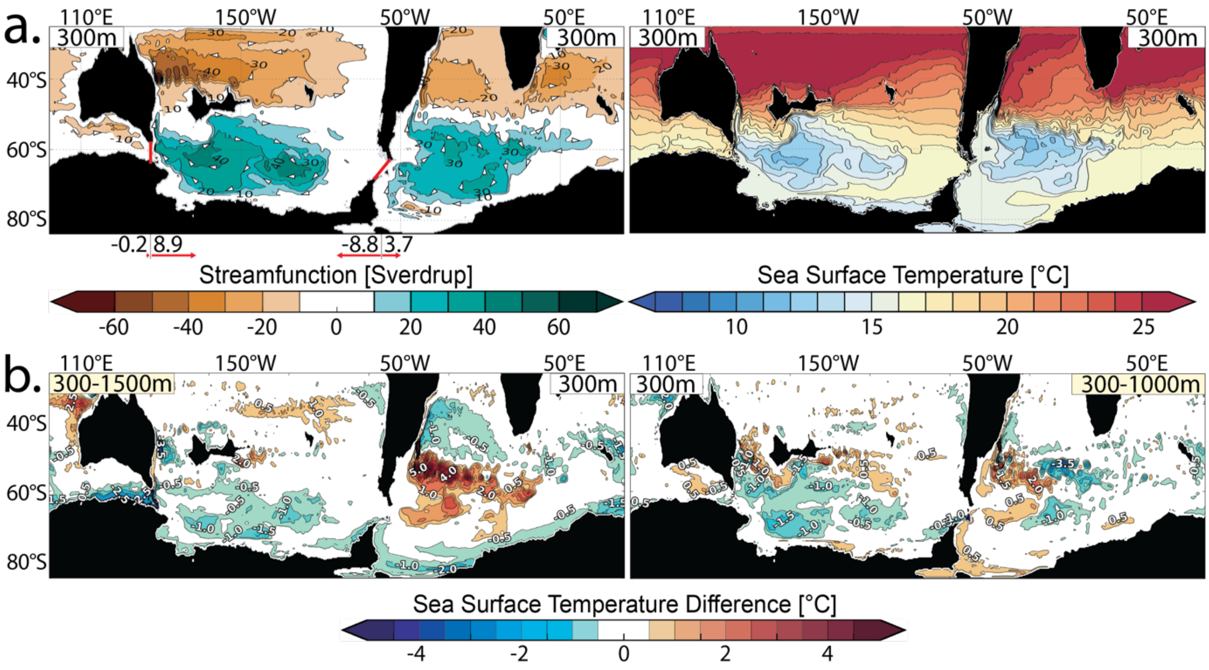

Supplementary Figure 6 Both Southern Ocean gateways are at shallow depth (300 m). **a.** Stream function and SSTs are shown (legend as in Figure 2), as well as **b.** the resulting SST differences after the deepening of the Tasmanian Gateway (TG) from 300 m to 1500 m (Drake Passage (DP) at 300 m) and after the deepening of the DP from 300 m to 1000 m (TG at 300 m).

2.1.2 Northern extension to the equator (Tasmanian Gateway: 300 m, Drake Passage: 1000 m)

We extended the northern boundary from 25°S to 0°S for the model with a shallow TG (300 m) and a deep DP (1000 m) in order to test the hypothesis of a "proto-ACC"-type current flowing north of Australia after the opening of a deep Drake Passage[(45)]. Such a flow has been detected in previous model simulations[(45)]. Although we observe a flow north of Australia through the open, wide "Indonesian seaway", the current forms a clockwise gyre and flows back westward through the Indonesian seaway (Supplementary Figure 7). The large-scale clockwise gyres remain dominant in the subpolar Atlantic and Pacific, showing similar transport values (>40 Sv) compared to the setup with the northern boundary at 25°S (Figure 2a). The Antarctic coast is kept similarly warm with SSTs reaching up to 19 °C. The net

transport through the Southern Ocean gateways are about 2% (TG) and 0.5% (DP) of today's ACC transport (170 Sv[42]).

A possible reason why our results differ from previous studies is the more complex bathymetry in the Pacific Ocean, containing a large mid ocean ridge system, as well as a complex continental Zealandia structure, both of which could act as a barrier for southward flow towards the DP. Higher resolution may be an additional reason for differences in the observed ocean dynamics.

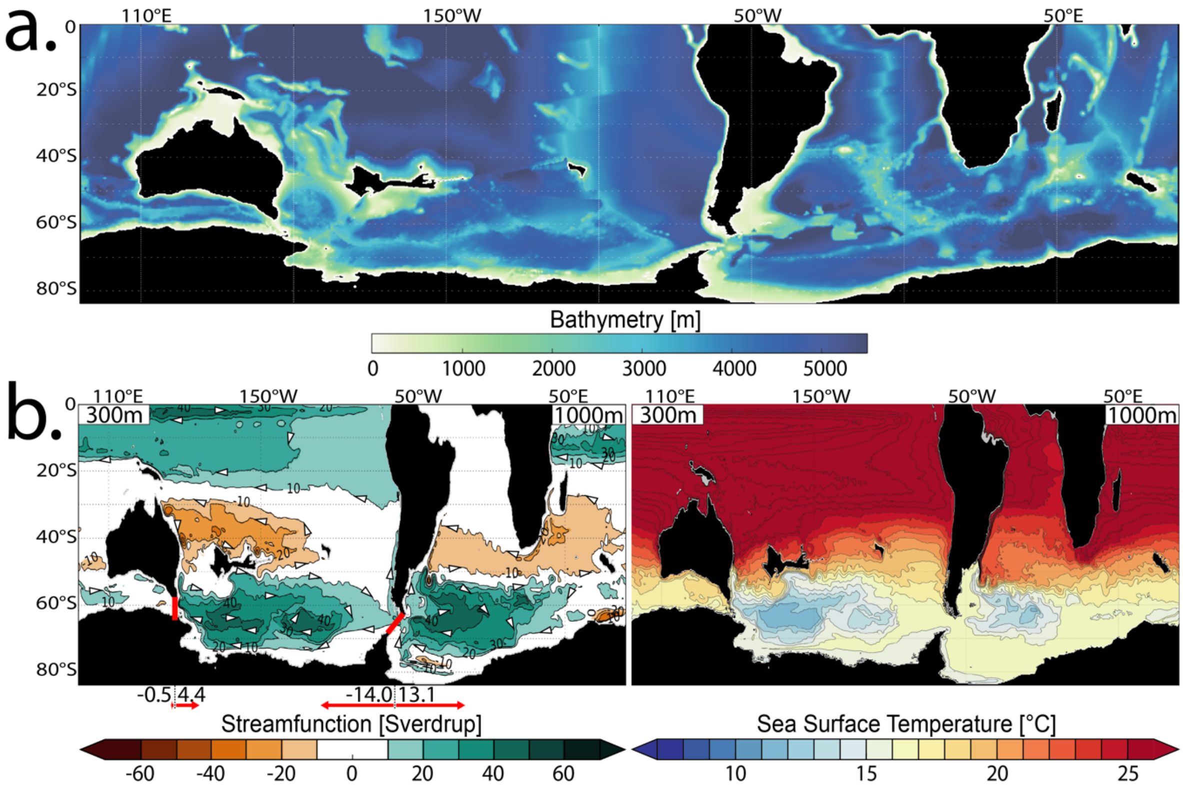

Supplementary Figure 7 Extension of the model's northern boundary to the equator, with a shallow Tasmanian Gateway (300 m) and deep Drake Passage (1000 m). **a.** Bathymetry, **b.** stream function and sea surface temperatures (legend as in Figure 2, main text).

2.1.3 Present-day bathymetry

This ocean simulation uses the present-day bathymetry ETOPO-1[41] with the same paleo atmospheric forcing as the other simulations presented in this study (Supplementary Figure 8). A prominent circumpolar current dominates the Southern Ocean flowing through wide and deep Southern Ocean gateways. However, the net transport through the gateway

reaches only about 22% of today's ACC transport and the ACC frontal systems are further south (modelled zonal wind stress has the maximum at 55°S) compared with today's ACC conditions. In addition, our model does not produce significantly strong gyres in the Weddell and Ross Sea, which reach about 60–65 Sv (Weddell Gyre[29]) and 15-30 Sv (Ross Gyre[31,32]) strength today. Furthermore, the SSTs offshore of Antarctica reach values of ~10 °C which is much warmer than today's conditions, but very close to the SST observation in the model run with both gateways below 600 m (Figure 2; Supplementary Figure 3).

These results lead us to suggest that it is the increasing equator-to-pole SST gradients that are responsible for the ACC to strengthen during post-EOT evolution until reaching its present-day strength of 170 Sv[42].

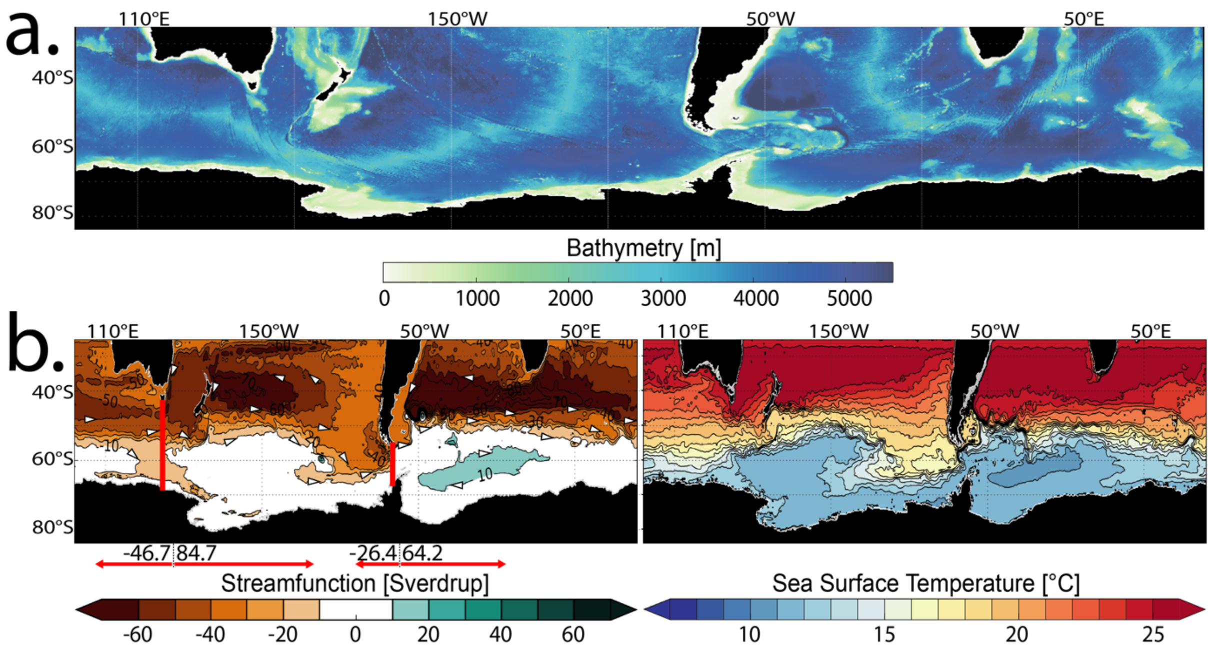

Supplementary Figure 8 Ocean model with present-day bathymetry (ETOPO-1[41]) with **a.** bathymetry, **b.** stream function and sea surface temperatures (legend as in Figure 2, main text).

2.1.4 Closed gateway (at 0 m)

We simulate the oceanographic consequences with one gateway entirely closed (TG at 0 m depth) and a deep second gateway (DP at 1000 m; Supplementary Figure 9a). With the early opening (from 0 m to 300 m), the subpolar gyres remain relatively stable in size; however, the

subpolar Pacific gyre decreases slightly in strength, from ~50 Sv to ~40 Sv. The observed SST changes are small, not exceeding 1 °C differences, during the initial, shallow opening of the second gateway (Supplementary Figure 9c). It requires a deepening to depths > 300 m to achieve significant surface water cooling along the Antarctic coast.

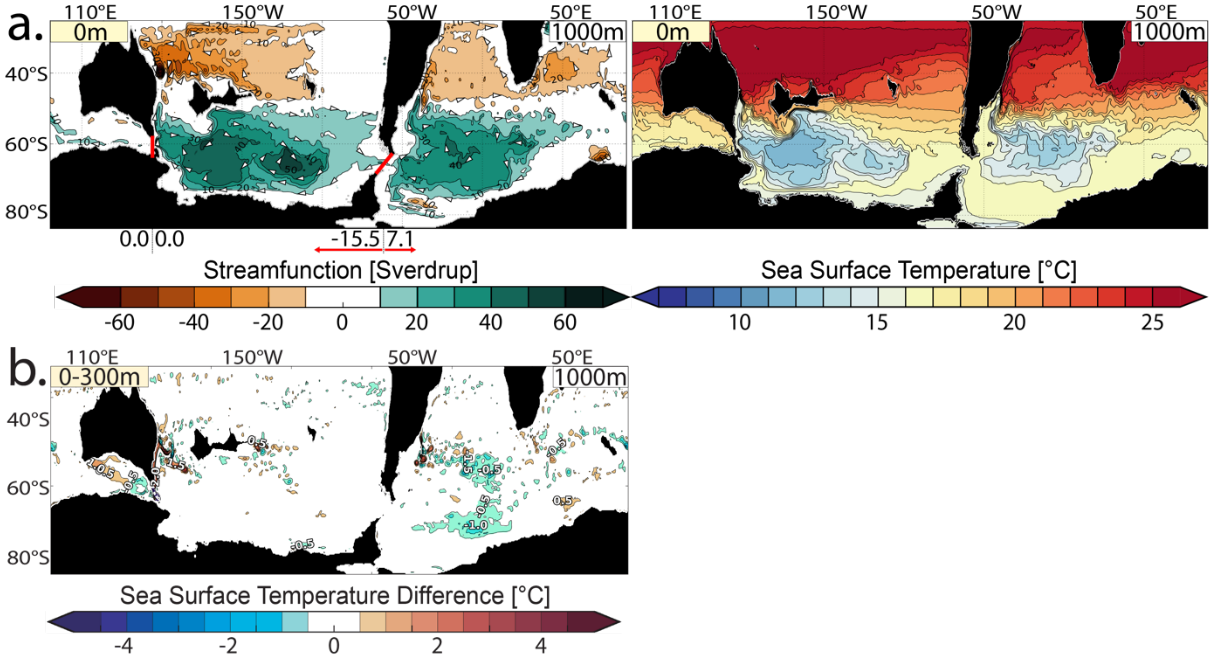

Supplementary Figure 9 Ocean simulation with Tasmanian Gateway (TG) being entirely closed (0 m depth) and Drake Passage at 1000 m. **a.** Stream function and SSTs are shown (legend as in Figure 2), **b.** SST changes due to TG deepening from 0 m to 300 m.

2.1.5 Changing model resolution and seafloor roughness

Four additional model simulations are run in order to investigate the impacts of changing ocean model resolution and bathymetry roughness on the ocean circulation pattern, sea surface temperature distribution, and the sensitivity to changing gateway depths in the Southern Ocean. For the representative model simulations, we set TG depth to 300 m and 1500 m and keep DP constant at 1000 m depth. For two simulations the horizontal resolution of the ocean model is set to 1°; this suppresses eddies in the Southern Ocean, and their transport is parameterised instead (section 2.1.5.1). The gateway depths have been manually adjusted to preserve the target depths, despite the coarser resolution (Supplementary Figure 10). In order to test the model sensitivity on seafloor roughness, two additional 0.25° runs are implemented using smoothed bathymetry grids (Supplementary Figure 11). These grids have

been smoothed to 3° resolution, in order to reduce the small-scale roughness, and re-gridded again to 0.25° (section 2.1.5.2). The gateway depths have been manually adjusted to preserve the target depths.

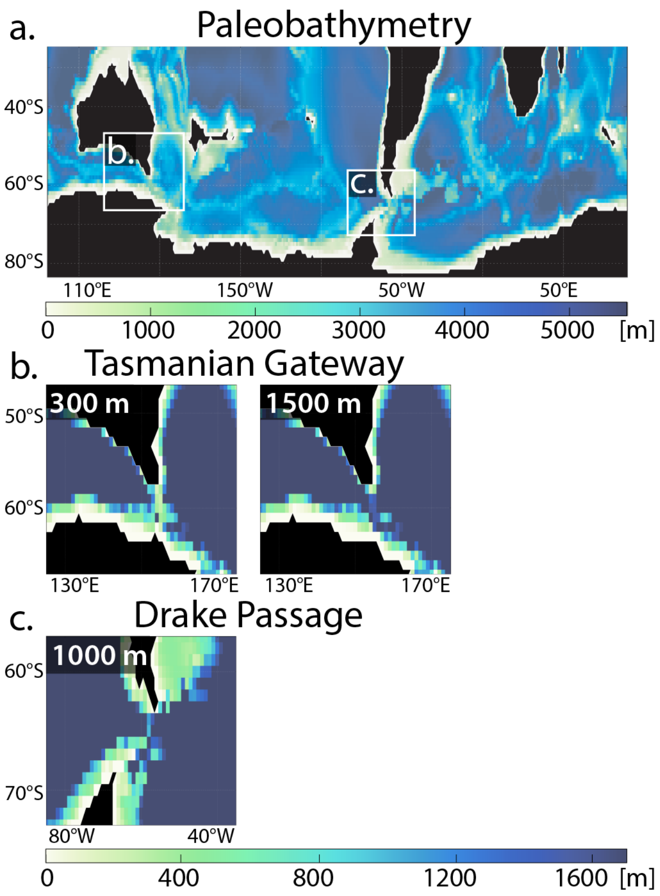

Supplementary Figure 10 Low-resolution (1°) bathymetry grids of **a.** the Southern Ocean with **b.** Tasmanian Gateway (TG) at 300 m and 1500 m, and **c.** DP at 1000 m.

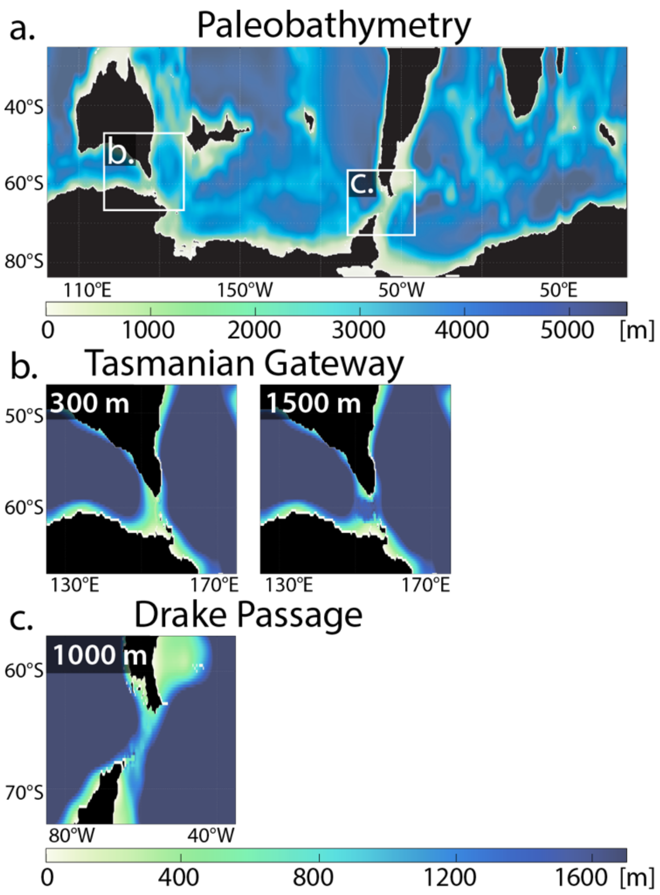

Supplementary Figure 11 Smoothed bathymetry grids of **a.** the Southern Ocean with **b.** Tasmanian Gateway (TG) at 300 m and 1500 m, and **c.** DP at 1000 m.

2.1.5.1 Low-resolution (1°) simulations

When one gateway is shallow (TG at 300 m), the subpolar gyres reach a strength of >20 Sv and the net transports through the gateways are 11.2 Sv (TG) and 3 Sv (DP) (Supplementary Figure 12a). Compared to the high-resolution 0.25° model results with the same depth configuration (Figure 2a), the 1° gyres reach only half the strength and size. The SSTs are remarkably different to the 0.25° run. Whereas the 0.25° simulation shows strong subpolar gyres transporting warm waters to the Antarctic coast and forming a cold deep-water upwelling centre (Figure 2a), the 1° model shows no significant southward transport of warm surface waters to the Antarctic coast (Supplementary Figure 12a). Instead, a stratified north-south SST gradient is observed reaching slightly colder SSTs along large parts of the Antarctic coast (absolute SSTs of 14–16 °C offshore West Antarctica) compared to the 0.25° equivalent (1–2 °C colder).

When TG deepens to 1500 m, the subpolar gyres shrink in size and reach strength of >10 Sv (Supplementary Figure 12b), whereas the net transports are 23.3 Sv (TG) and 10.5 Sv (DP). In comparison, the 0.25° subpolar Pacific gyre remains three times as strong as the 1° equivalent, however, the net transport is also stronger in the 0.25° run (31.3 Sv through TG, 12.5 Sv through DP; Figure 2c). Whilst the 0.25° simulation with two deep SO gateways show SSTs offshore Antarctica of ~11 °C (Figure 2c), the SSTs in the 1° run remain at ~14 °C (Supplementary Figure 12b). The difference between the gyre strengths in the coarse and fine resolution experiments could reflect either driving of the gyres by eddies, or suppression of the gyres due to the parameterized mixing in the coarse runs. Further analysis of this is underway.

The most prominent consequence of coarsening the model resolution is that the 1° SST distribution in the Southern Ocean does not change significantly with gateway deepening and remains relatively stable (Supplementary Figure 12c). Whereas the Antarctic coast in the Indian sector cools down slightly (average decrease by 0.5–1 °C), the Pacific and Atlantic sectors warm slightly (average increase by 0.5–1 °C, maximum of 1.5 °C; Supplementary Figure 12c). The observed small SST changes are in line with previous studies, indicating similarly little cooling of Antarctic surface waters in response to changes in the gateways[e.g.46]. It stands in stark contrast however to the 0.25° simulation which exhibits an average of 2–4 °C and up to 5 °C cooling along the Antarctic coast with TG deepening from 300 m to 1500 m (Supplementary Figure 4).

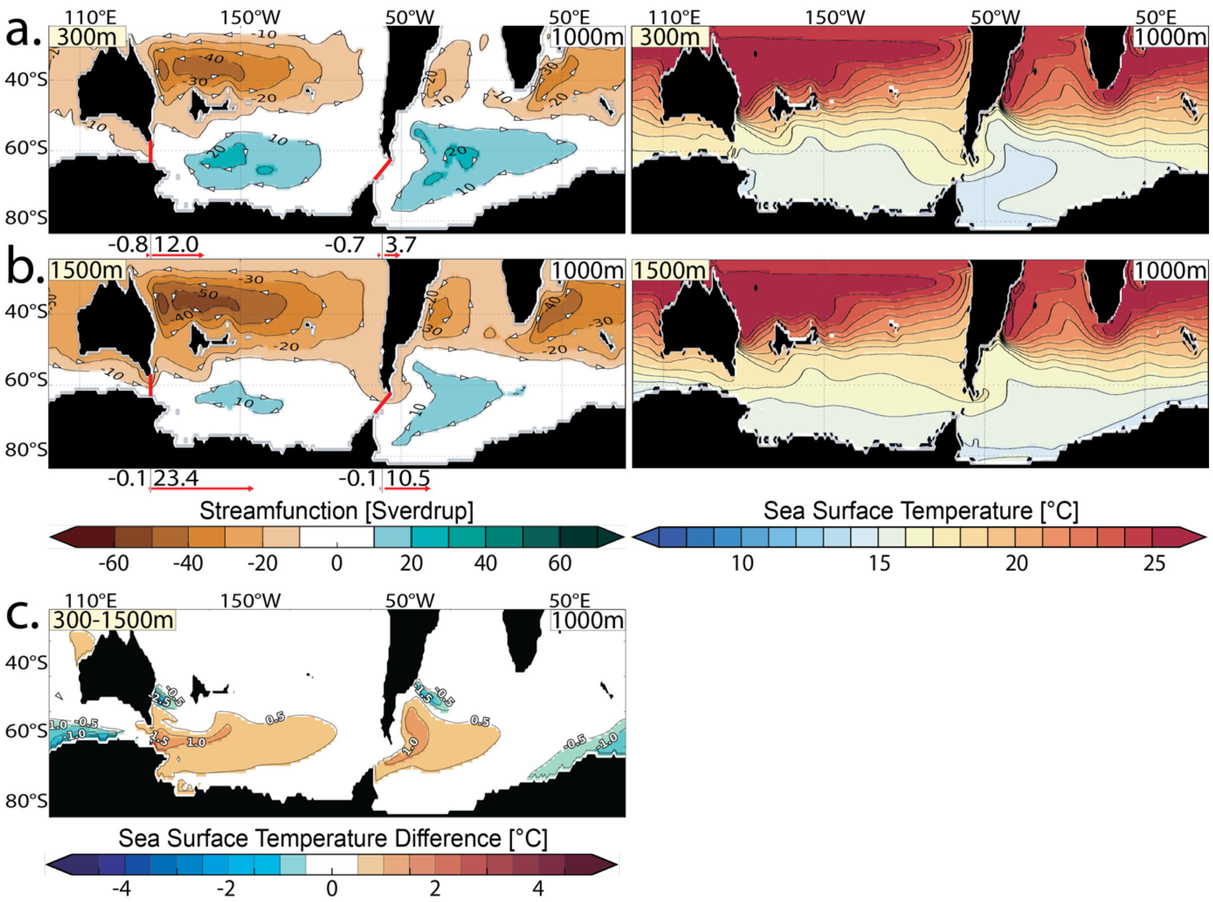

Supplementary Figure 12 Sensitivity model runs with 1° ocean model resolution (resulting bathymetry grid, see Supplementary Figure 10). Stream function and SST are shown with TG at **a.** 300 m, and **b.** 1500 m (DP at 1000 m; legend as in Figure 2), **c.** shows the resulting sea surface temperature change with TG deepening from 300 m to 1500 m.

2.1.5.2 Smooth bathymetry

The 0.25° model run using smooth bathymetry with a 300 m deep TG yields a subpolar Atlantic gyre similar in strength and size to that in the "rough" counterpart. In addition, a very strong gyre centre in the southeast Pacific is observed (reaching 90 Sv; Supplementary Figure 13a). This occurs in a region of closed "f/H contours" and is likely eddy-driven[(e.g.46,47)]. In the subpolar Atlantic the SSTs offshore Antarctica are about 13–14 °C, which is 3–4 °C colder than the SSTs in the "rough" simulation (Figure 2a). Furthermore, the Antarctic coast along the Southwest Pacific reaches slightly colder temperatures with the smooth bathymetry. However, no significant differences in the Australian-Antarctic SST distributions can be observed between the "rough" and "smooth" run. The "smooth" net transport through the

300 m TG and 1000 m DP are 15.6 Sv and 5.1 Sv, respectively, which is stronger in eastward direction compared to the “rough” equivalent (~3.2 Sv and -6.2 Sv; Figure 2a).

When TG deepens to 1500 m, the gyres weaken (although, the subpolar Pacific one remains >60 Sv). The “smooth” gyres are about twice as strong as their “rough” counterpart. The “smooth” SSTs offshore Antarctica show a cooling trend, however, not as much as in the “rough” run (Supplementary Figure 13b). The “smooth” net transport reaches similar values to the “rough” equivalent (TG: 33.5 Sv and DP: 17.9 Sv).

The consequences of gateway deepening are still visible with weakening and shrinking subpolar gyres and Antarctic surface water cooling (Supplementary Figure 13c). However, the cooling is reduced compared to model runs with rough seafloor (Supplementary Figure 4). In the Indian and Atlantic sectors of the Antarctic coast, the surface waters cool on average by 2–2.5 °C, whereas the Pacific sector experiences no SST change directly offshore Antarctica, however, SSTs warm up within the subpolar Pacific gyre region and along the eastern Australian coast (average of 1–2.5 °C, maximum of 3.5 °C; Supplementary Figure 13c).

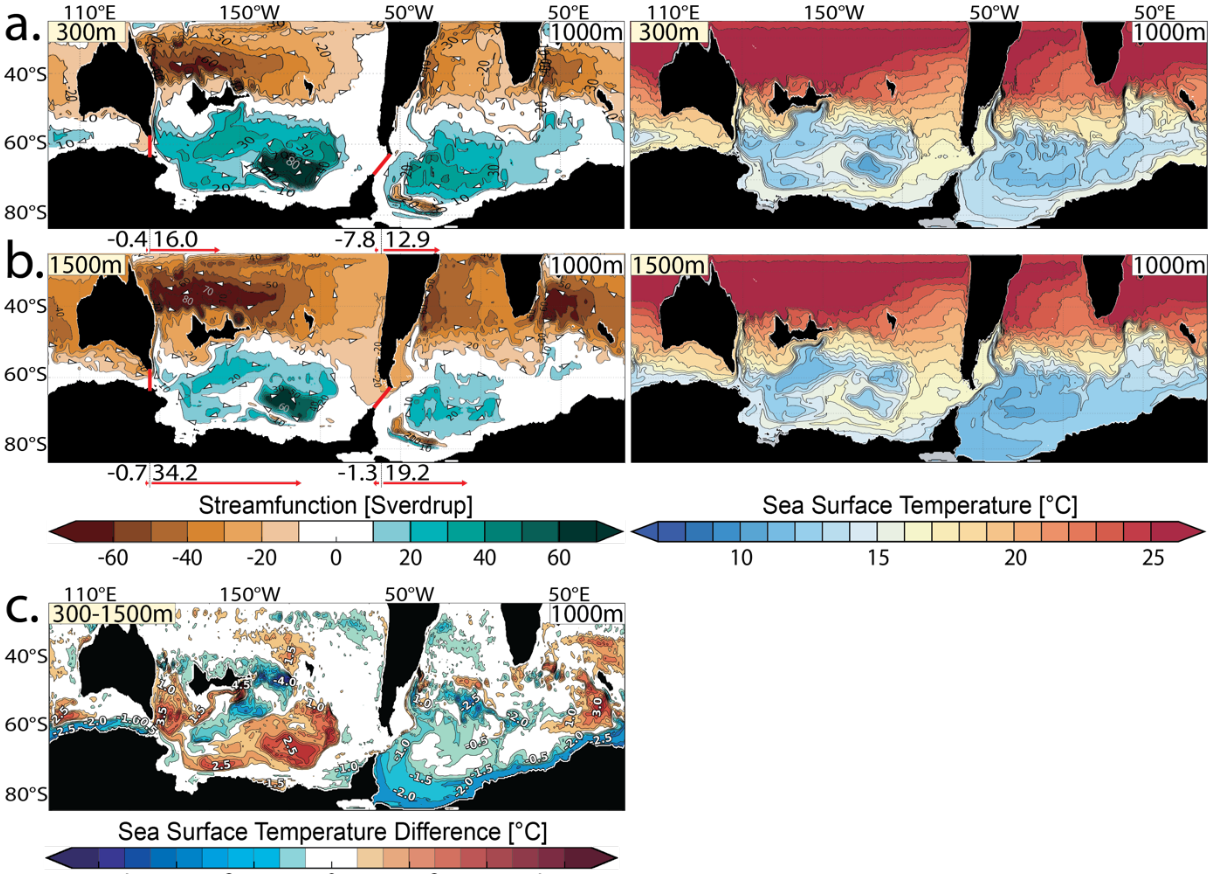

Supplementary Figure 13 Sensitivity model runs using a smooth bathymetry grid (see Supplementary Figure 11). Stream function and SST are shown with TG at **a.** 300 m, and **b.** 1500 m (DP at 1000 m; legend as in Figure 2), **c.** shows the resulting sea surface temperature change with TG deepening from 300 m to 1500 m.

It can be concluded that both improved ocean model resolution and the presence of detailed seafloor roughness are crucial for the strong SST cooling and changes in the ocean circulation dynamics to reconstruct in the Southern Ocean.

Supplementary references

[1] Schouten, Stefan, et al. "Distributional variations in marine crenarchaeotal membrane lipids: a new tool for reconstructing ancient sea water temperatures?" *Earth and Planetary Science Letters* 204.1-2 (2002): 265-274.

[2] Brassell, S. C., et al. "Palaeoclimatic signals recognized by chemometric treatment of molecular stratigraphic data." *Organic Geochemistry* 10.4-6 (1986): 649-660.

[3] Prahl, Fred G., and Stuart G. Wakeham. "Calibration of unsaturation patterns in long-chain ketone compositions for palaeotemperature assessment." *Nature* 330.6146 (1987): 367.

[4] Bijl, Peter K., et al. "Early Palaeogene temperature evolution of the southwest Pacific Ocean." *Nature* 461.7265 (2009): 776.

[5] Sluijs, Appy, et al. "Arctic late Paleocene–early Eocene paleoenvironments with special emphasis on the Paleocene-Eocene thermal maximum (Lomonosov Ridge, Integrated Ocean Drilling Program Expedition 302)." *Paleoceanography* 23.1 (2008).

[6] Schouten, Stefan, Ellen C. Hopmans, and Jaap S. Sinninghe Damsté. "The organic geochemistry of glycerol dialkyl glycerol tetraether lipids: a review." *Organic geochemistry* 54 (2013): 19-61.

[7] Sluijs, Appy, et al. "A middle Eocene carbon cycle conundrum." *Nature Geoscience* 6.6 (2013): 429-434.

[8] Cramwinckel, Margot J., et al. "Surface-circulation change in the southwest Pacific Ocean across the Middle Eocene Climatic Optimum: inferences from dinoflagellate cysts and biomarker paleothermometry." *Climate of the Past* 16.5 (2020): 1667-1689.

[9] Bijl, P. K., Houben, A. J. P., Schouten, S., Bohaty, S. M., Sluijs, A., Reichart, G.-J., Damsté, J. S. S., and Brinkhuis, H.: Transient Middle Eocene Atmospheric CO2 and Temperature Variations, *Science*, 330, 819–821 (2010).

[10] Bijl, Peter K., et al. "Eocene cooling linked to early flow across the Tasmanian Gateway." *Proceedings of the National Academy of Sciences* 110.24 (2013): 9645-9650.

[11] Inglis, Gordon N., et al. "Descent toward the Icehouse: Eocene sea surface cooling inferred from GDGT distributions." *Paleoceanography* 30.7 (2015): 1000-1020.

[12] Douglas, Peter MJ, et al. "Pronounced zonal heterogeneity in Eocene southern high-latitude sea surface temperatures." *Proceedings of the National Academy of Sciences* 111.18 (2014): 6582-6587.

[13] Houben, Alexander JP, et al. "Late Eocene Southern Ocean cooling and invigoration of circulation preconditioned Antarctica for full-scale glaciation." *Geochemistry, Geophysics, Geosystems* (2019).

[14] Hollis, Christopher J., et al. "Early Paleogene temperature history of the Southwest Pacific Ocean: Reconciling proxies and models." *Earth and Planetary Science Letters* 349 (2012): 53-66.

[15] Liu, Zhonghui, et al. "Global cooling during the Eocene-Oligocene climate transition." *Science* 323.5918 (2009): 1187-1190.

[16] Plancq, Julien, et al. "Productivity and sea-surface temperature changes recorded during the late Eocene–early Oligocene at DSDP Site 511 (South Atlantic)." *Palaeogeography, Palaeoclimatology, Palaeoecology* 407 (2014): 34-44.

[17] Hartman, Julian D., Francesca Sangiorgi, and Carlota Escutia. "Paleoceanography and ice sheet variability offshore Wilkes Land, Antarctica–Part 3: Insights from Oligocene–Miocene TEX86-based sea surface temperature reconstructions." *Climate of the Past 14.7* (2018): 1015-1033.

[18] Hoem, Frida S., et al. "Temperate Oligocene surface ocean conditions offshore Cape Adare, Ross Sea, Antarctica." *Climate of the Past Discussions* (2020): 1-32.

[19] Tibbett, Emily J., et al. "Late Eocene record of hydrology and temperature from Prydz Bay, East Antarctica." *Paleoceanography and Paleoclimatology* (2021): e2020PA004204.

[20] Prebble, J. G., et al. "An expanded modern dinoflagellate cyst dataset for the Southwest Pacific and Southern Hemisphere with environmental associations." *Marine Micropaleontology* 101 (2013): 33-48.

[21] Esper, Oliver, and Karin AF Zonneveld. "Distribution of organic-walled dinoflagellate cysts in surface sediments of the Southern Ocean (eastern Atlantic sector) between the Subtropical Front and the Weddell Gyre." *Marine Micropaleontology* 46.1-2 (2002): 177-208.

[22] Zonneveld, Karin AF, et al. "Atlas of modern dinoflagellate cyst distribution based on 2405 data points." *Review of Palaeobotany and Palynology* 191 (2013): 1-197.

[23] Houben, Alexander JP, et al. "Reorganization of Southern Ocean plankton ecosystem at the onset of Antarctic glaciation." *Science* 340.6130 (2013): 341-344.

[24] Sangiorgi, Francesca, et al. "Southern Ocean warming and Wilkes Land ice sheet retreat during the mid-Miocene." *Nature communications* 9.1 (2018): 317.

[25] Eynaud, F., et al. "Sea-surface distribution of coccolithophores, diatoms, silicoflagellates and dinoflagellates in the South Atlantic Ocean during the late austral summer 1995." *Deep Sea Research Part I: Oceanographic Research Papers* 46.3 (1999): 451-482.

[26] Sluijs, Appy, Jörg Pross, and Henk Brinkhuis. "From greenhouse to icehouse; organic-walled dinoflagellate cysts as paleoenvironmental indicators in the Paleogene." *Earth-Science Reviews* 68.3-4 (2005): 281-315.

[27] Frieling, Joost, and Appy Sluijs. "Towards quantitative environmental reconstructions from ancient non-analogue microfossil assemblages: Ecological preferences of Paleocene–Eocene dinoflagellates." *Earth-Science Reviews* 185 (2018): 956-973.

[28] Bijl, Peter K., et al. "Environmental forcings of Paleogene Southern Ocean dinoflagellate biogeography." *Paleoceanography* 26.1 (2011).

[29] Schröder, Michael, and Eberhard Fahrbach. "On the structure and the transport of the eastern Weddell Gyre." *Deep Sea Research Part II: Topical Studies in Oceanography* 46.1-2 (1999): 501-527.

[30] Nooteboom, Peter D., et al. "Transport bias by ocean currents in sedimentary microplankton assemblages: Implications for paleoceanographic reconstructions." *Paleoceanography and Paleoclimatology* 34.7 (2019): 1178-1194.

[31] Mazloff, Matthew R., Patrick Heimbach, and Carl Wunsch. "An eddy-permitting Southern Ocean state estimate." *Journal of Physical Oceanography* 40.5 (2010): 880-899.

[32] Nakayama, Yoshihiro, et al. "Modeling the spreading of glacial meltwater from the Amundsen and Bellingshausen Seas." *Geophysical Research Letters* 41.22 (2014): 7942

[33] González-Estebenet, M. Sol, G. Raquel Guerstein, and Martín E. Rodriguez Raising. "Middle Eocene Dinoflagellate cysts from Santa Cruz Province, Argentina: biostratigraphy and paleoenvironment." *Review of Palaeobotany and Palynology* 211 (2014): 55-65.

[34] Frieling, Joost, et al. "Extreme warmth and heat-stressed plankton in the tropics during the Paleocene-Eocene Thermal Maximum." *Science advances* 3.3 (2017): e1600891.

[35] Cramwinckel, Margot J., et al. "Synchronous tropical and polar temperature evolution in the Eocene." *Nature* 559.7714 (2018): 382.

[36] Scher, H. D. et al. "Onset of Antarctic Circumpolar Current 30 million years ago as Tasmanian Gateway aligned with westerlies." *Nature* 523, 580–583 (2015).

[37] Goldstein, Steven L., and Sidney R. Hemming. "Long-lived isotopic tracers in oceanography, paleoceanography, and ice-sheet dynamics." *Treatise on geochemistry* 6 (2003): 625.

[38] Ling, H. F., et al. "Evolution of Nd and Pb isotopes in Central Pacific seawater from ferromanganese crusts." *Earth and Planetary Science Letters* 146.1-2 (1997): 1-12.

[39] Thomas, Deborah J., Timothy J. Bralower, and Charles E. Jones. "Neodymium isotopic reconstruction of late Paleocene–early Eocene thermohaline circulation." *Earth and Planetary Science Letters* 209.3-4 (2003): 309-322.

[40] Scher, Howie D., and Ellen E. Martin. "Timing and climatic consequences of the opening of Drake Passage." S*cience* 312.5772 (2006): 428-430.

[41] Amante, Christopher, and Barry W. Eakins. "ETOPO1 arc-minute global relief model: procedures, data sources and analysis." *NOAA Technical Memorandum NESDIS*, NGDC-24 (2019): 19 pp.

[42] Donohue, K. A., et al. "Mean Antarctic Circumpolar Current transport measured in Drake Passage." *Geophysical Research Letters* 43.22 (2016): 11-760.

[43] Gulick, Sean PS, et al. "Initiation and long-term instability of the East Antarctic Ice Sheet." *Nature* 552.7684 (2017): 225-229.

[44] Carter, Andrew, et al. "Widespread Antarctic glaciation during the late Eocene." *Earth and Planetary Science Letters* 458 (2017): 49-57.

[45] Sijp, Willem P., Anna S. von der Heydt, and Peter K. Bijl. "Model simulations of early westward flow across the Tasman Gateway during the early Eocene." *Climate of the Past* 12.4 (2016): 807-817.

[46] Huber, M. et al. "Eocene circulation of the Southern Ocean: was Antarctica kept warm by subtropical waters?" *Paleoceanography* 19 (2004).

[46] Bretherton, Francis P., and Dale B. Haidvogel. "Two-dimensional turbulence above topography." *Journal of Fluid Mechanics* 78.1 (1976): 129-154.

[47] LaCasce, J. H., O. A. Nøst, and P. E. Isachsen. "Asymmetry of free circulations in closed ocean gyres." *Journal of Physical Oceanography* 38.2 (2008): 517-526.
